# Chimeric antigen receptor T-cell therapy for multiple myeloma

**DOI:** 10.3389/fimmu.2022.1050522

**Published:** 2022-12-22

**Authors:** Zehua Wang, Chen Chen, Lei Wang, Yongxu Jia, Yanru Qin

**Affiliations:** Department of Oncology, The First Affiliated Hospital of Zhengzhou University, Zhengzhou, China

**Keywords:** multiple myeloma, immunotherapy, chimeric antigen receptor cell therapy, resistance mechanism, efficacy, toxicity

## Abstract

Multiple myeloma (MM) is a malignant plasma cell disorder that remains incurable for most patients, as persistent clonal evolution drives new mutations which confer MM high-risk signatures and resistance to standard care. The past two decades have significantly refashioned the therapeutic options for MM, especially adoptive T cell therapy contributing to impressive response rate and clinical efficacy. Despite great promises achieved from chimeric antigen receptor T-cell (CAR-T) therapy, the poor durability and severe toxicity (cytokine release syndrome and neurotoxicity) are still huge challenges. Therefore, relapsed/refractory multiple myeloma (RRMM), characterized by the nature of clinicopathologic and molecular heterogeneity, is frequently associated with poor prognosis. B Cell Maturation Antigen (BCMA) is the most successful target for CAR-T therapy, and other potential targets either for single-target or dual-target CAR-T are actively being studied in numerous clinical trials. Moreover, mechanisms driving resistance or relapse after CAR-T therapy remain uncharacterized, which might refer to T-cell clearance, antigen escape, and immunosuppressive tumor microenvironment. Engineering CAR T-cell to improve both efficacy and safety continues to be a promising area for investigation. In this review, we aim to describe novel tumor-associated neoantigens for MM, summarize the data from current MM CAR-T clinical trials, introduce the mechanism of disease resistance/relapse after CAR-T infusion, highlight innovations capable of enhanced efficacy and reduced toxicity, and provide potential directions to optimize manufacturing processes.

## 1 Introduction

Multiple myeloma (MM) is a malignant plasma cell disorder that displays a myriad of manifestations including hypercalcemia, renal insufficiency, anemia, and bone destruction (CRAB) (1, 2). MM is the second most common hematological malignancy with an estimated 32270 new cases and 12830 deaths in the United States in 2020 (3). Genetic abnormities, mostly translocation and hyper-diploidy, result in dysregulated cancer-immunity cycle that allows MM to escape immune surveillance with an uncontrolled cell proliferation (4, 5). The past two decades have significantly refashioned the therapeutic options of MM, such as the availability of proteasome inhibitors (PI), immunomodulatory drugs (IMiDs), histone deacetylase inhibitors (HDACi), anti-CD38 monoclonal antibodies (mABs), antibody-drug conjugates (ADC), and selective inhibitors nuclear export (SINE) (6). However, MM remains incurable for most patients, as persistent clonal evolution drives new mutations which confer MM high-risk signatures and resistance to standard care (7, 8). Therefore, relapsed/refractory multiple myeloma (RRMM), characterized by the nature of clinicopathologic and molecular heterogeneity (9, 10), is frequently associated with poor prognosis (11).

**Figure 1:**
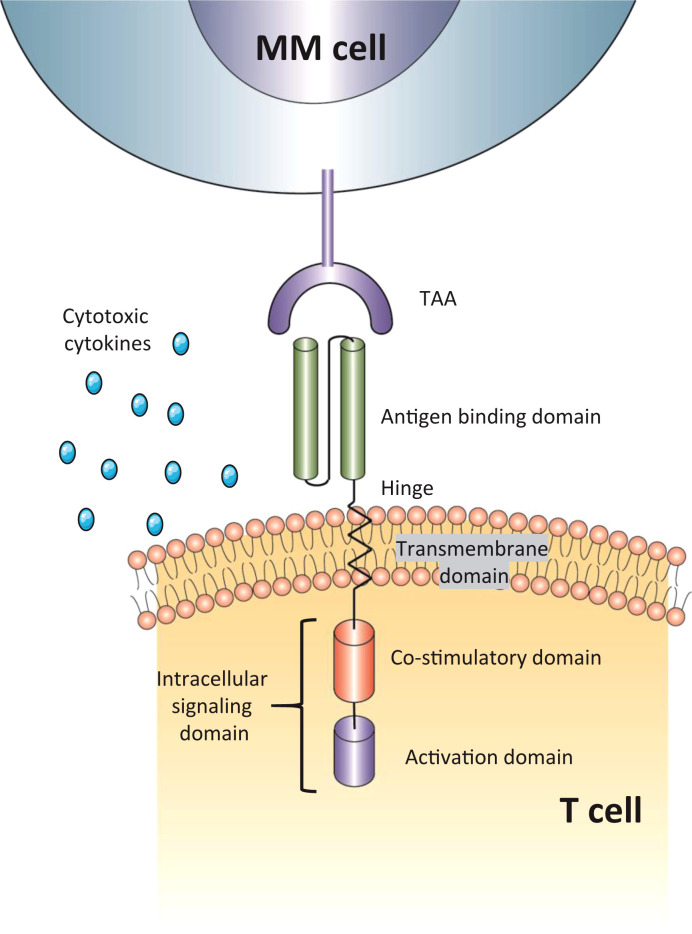
The structure of CAR-T cells. The antigen-binding domain is usually a single-chain variable fragment (scFv) but also has other structures; intracellular signaling domain include both co-stimulatory domain (CD28, 4-1BB) and activation domain (CD3zeta). TAA, tumor-associated antigen; MM cell, multiple myeloma cell.

Chimeric antigen receptor T-cell therapy (CAR-T) has shown exceptional success in the treatment of relapsed/refractory B-cell acute lymphoblastic leukaemia (B-ALL), B-cell chronic lymphoblastic leukaemia (B-CLL), and diffuse large B-cell Lymphoma (DLBCL) (12, 13), thereby motivating its application in RRMM (14). T cells are firstly isolated from the patients’ or donors’ blood and genetically modified in the laboratory to encode an artificial receptor, enabling CAR T cells to identify targets better and precisely destroy cancer cells. CAR T-cell functions with two major roles: 1) tumor-associated antigen (TAA) binding; 2) MHC-independent T-cell activation. Emerging as a novel immunotherapy, CAR T-cell therapy consists of an extracellular antigen recognition domain (scFv, Fab, Nb, and NKG2D ligand), a transmembrane domain, and an intracellular domain incorporating co-stimulation (CD28 or a 4-1BB) and signaling components (CD3zeta) (**Figure 1**) (15, 16). The interplay between tumor cell and CAR gives rise to an immunological synapse. This process could attack target cells through various pathways, such as the release of cytotoxic molecules, and the induction of apoptosis signal pathway, eventually leading to the activation of effector T cells and elimination of tumor cells (17).

Despite great promises achieved by CAR-T therapy, the poor durability and severe toxicity are still huge challenges. The mechanisms driving resistance and relapse after MM CAR T-cell therapy remain uncharacterized. Consequently, this review aims to describe candidate tumor-associated neoantigens for MM, provide a summary of efficacy and safety data from clinical trials, introduce mechanisms of disease resistance/relapse to CAR-T, and explore future innovations capable of enhanced efficacy and reduced toxicity, and provide potential directions to optimize manufacturing processes.

## 2 Candidate targets for multiple myeloma CAR-T

**Figure 2:**
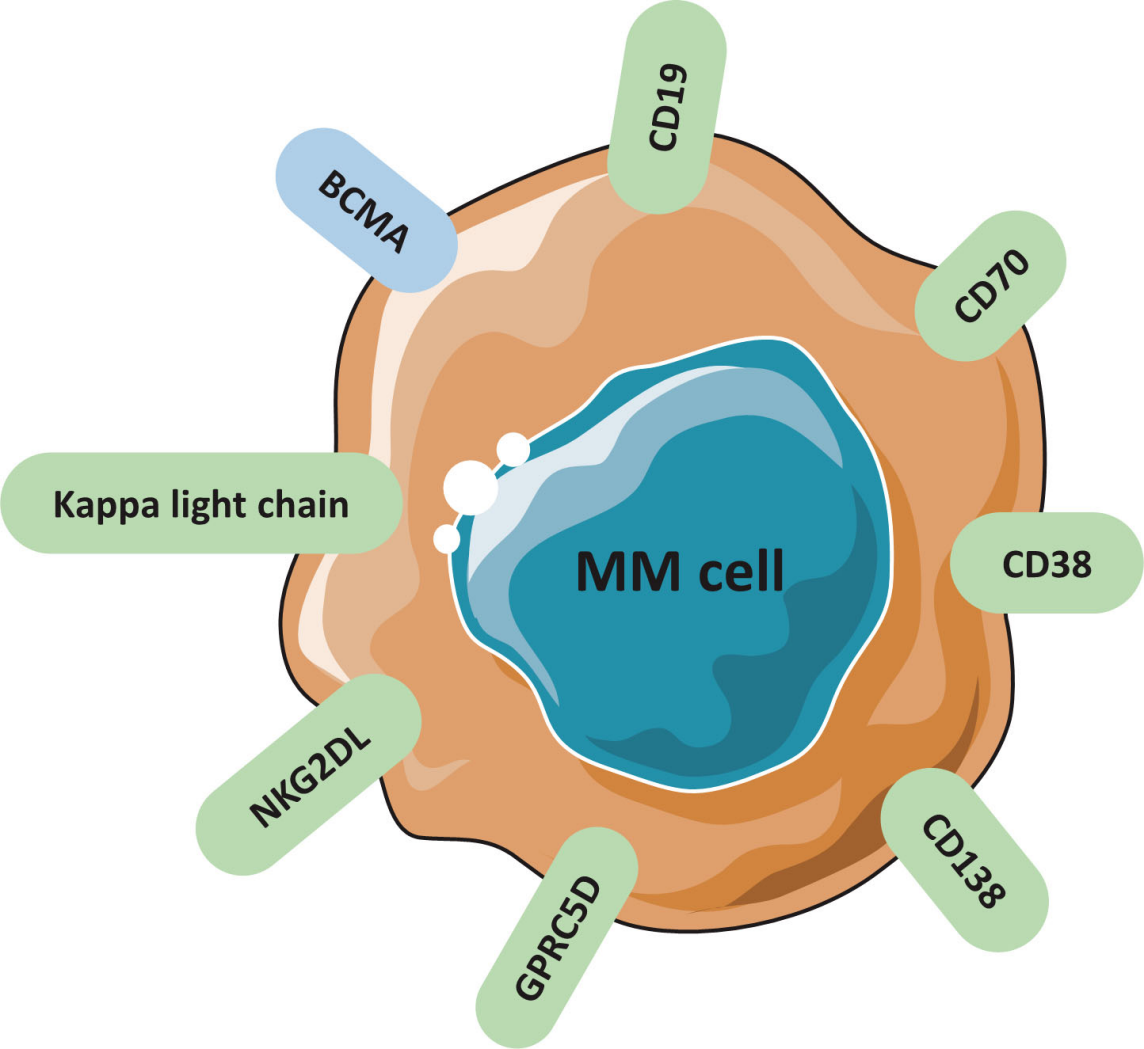
Candidate surface antigens found on MM cells and studied in clinical trials.

The key to design a successful CAR is to select a surface antigen that presents at high concentration on MM cells, but absent in non-malignant hematopoietic lineages or other tissues (18–21). The most important avenue is to discover novel TAAs to improve CAR-T therapy. Several targetable antigens are currently being evaluated regarding their safety and efficacy in clinical trials (**Tables 1**, **2**). Potential targetable antigens for MM are summarized in **Figure 2**, including BCMA, CD19, SLAMF7, GPRC5D, CD138, CD38, CD70, NKG2DL, Kappa light chain.

**Table 1:** Selected BCMA-targeted CAR-T clinical trials for MM.

Identifier	Target	Status	Phase	Enrollment	Study Population	Efficacy		Safety		Reference
						ORR (%)	Median PFS (month)	Grade > 3 CRS (%)	Grade > 3 NTX (%)	
NCT02215967	BCMA	Completed	I	24	RRMM	81	7.75	25	4	(22)
NCT02546167	BCMA	Completed	I	25	HRMM	48	2.7	32	12	(23)
NCT02658929	BCMA	Active	I	67	RRMM	76	8.8	6	3	(24)
NCT03274219	BCMA	Active	I	72	RRMM	55	11.9	4	6	(25)
NCT03975907	BCMA	Recruiting	I	62	RRMM	87.5	18.8	6	3	(26)
NCT03302403	BCMA	Active	I	18	RRMM	87.5	unknown	0	4	(27)
NCT03093168	BCMA	Unknown	I	10	RRMM	86	unknown	0	0	(28)
NCT04322292	BCMA	Recruiting	I	10	RRMM	95.2	unknown	5	0	(29)
NCT03661554	BCMA	Unknown	I	15	RRMM	88.2	12.1	2.9	0	(30)
NCT03090659	BCMA	Active	I-II	74	RRMM	87.8	18.04	9.5	0	(31)
NCT03548207	BCMA	Active	I-II	97	RRMM	96.9	unknown	4.1	9.3	(32)
NCT03716856	BCMA	Active	I	24	RRMM	87.5	unknown	0	4.2	(26, 33)

**Abbreviations:** RRMM, relapsed or refractory multiple myeloma; HRMM, high risk multiple myeloma; CRS, cytokine release syndrome; NTX, neurotoxicity; ORR, overall response rate; PFS, progression-free survival.

**Table 2:** Selected non-BCMA-targeted CAR-T clinical trials for MM.

Neoantigen	Expression on MM cells	Expression on hematopoietic cells	Expression on other cells	Identifier	Status	Phase	Enrollment	Efficacy	Safety
CD19	weak expression	B-cell lineage cells	absent	NCT02135406	completed	I	10	ORR: 80%	AE: 0%
SLAMF7	increased expression	NK cells, T cells, B cells, dendritic cells, monocytes, macrophages	absent	NCT03958656	completed	I	13	NA	NA
GPRC5D	high expression	B cells and plasma cells	epithelial cells	NCT04555551	active	I	17	ORR: 83%	G3+ CRS: 8%
CD138	high expression	Plasma cells	epithelial cells	NCT01886976	recruiting	I-II	10	ORR: 80%	AE: 0%
CD38	increased expression	NK cells, T cells, dendritic cells, neutrophils, and progenitor cells	epithelial cells	NCT03464916	active	I	72	NA	NA
CD70	increased expression	germinal center B cells, T cells	stromal cells of the thymic medulla	NCT04662294	recruiting	I	108	NA	NA
NKG2D	increased expression	NK cells, T cells	absent	NCT02203825	completed	I	12	NA	AE: 0%

MM, multiple myeloma; ORR, overall response rate; AE, adverse event; CRS, cytokine release syndrome; G3+, Grade 3-4; NA, not available.

### 2.1 BCMA

B cell maturation antigen (BCMA), a transmembrane glycoprotein belonging to tumor necrosis factor (TNF) receptor superfamily, is the most commonly used surface antigen target for multiple myeloma CAR-T. BCMA plays a critical role in differentiating B-cell to plasma cell and maintaining the survival of plasma cell (34, 35). BCMA is preferentially expressed on plasma cells, though limited BCMA-positive cells can be identified in normal tissues, such as the spleen, lymph nodes, and the stomach (36, 37). A European study involving 70 MM patients identified that surface BCMA expression on plasma cells (normal or malignant) was significantly higher (P<0.001) than non-plasma cells (38). The high expression of surface BCMA is associated with MM in several preclinical models and humans, making it an attractive target for MM (39–41). However, BCMA could be expressed at high or low concentrations in MM cells (36, 42, 43). In a United Kingdom study, 28 evaluable MM patients all expressed BCMA, and levels differed from low to moderate (42). Similarly, a UK study reported that all 64 patients with MM expressed surface BCMA at varying levels by immunohistochemistry (43). Since there is a considerable variation in BCMA expression on MM cells, patients may respond differently to BCMA-targeted CAR-T therapy. As surface BCMA level may serve as an independent prognostic factor, cytogenetic assessments are of great importance (43). It is anticipated that patients with high levels of BCMA may gain more benefits from BCMA-targeted CAR-T therapy. Thereby, all findings support that BCMA may be a promising target for MM CAR-T therapy.

The first BCMA-targeted MM CAR-T clinical trial was conducted by National Cancer Institute (NCT02215967) (44). A total of 24 patients with RRMM were enrolled. The notable findings of this study were the dose-dependence of efficacy and toxicity. The ORR was 20% among 10 patients receiving the lowest dose of 0.3-3.0 × 10^6^ CAR-T cells/kg. However, of 16 patients treated with high-dose level, the ORR was 81% with 62.5% having very good partial response (VEGF) or better. Notably, the toxicity of low-dose CAR-T was generally modest and no patient with grade 3 or 4 cytokine release syndrome (CRS). By contrast, grade 3-4 CRS and neurotoxicity (NTX) were 25% and 4% among patients treated with highest dose (9 × 10^6^ CAR-T cells/kg). Further, a statistically significant relationship (P = 0.04) between plasma cell burden and severe CRS had been reported from patients with high-dose level of CAR-T cells. Many BCMA-targeted CAR-T clinical trials are ongoing or completed (**Table 1**). Additionally, combination therapies are evaluated as well, such as associating BCMA CARs with tyrosine kinase inhibitor (NCT04603827), immunomodulators (NCT03070327, NCT04287660), nonspecific immune inhibitors (NCT03943472), and gamma-secrete inhibitor (NCT03502577).

### 2.2 Non-BCMA targets

Though a majority of MM CAR-T clinical trials target BCMA, but there are several studies focused on non-BCMA MM-associated neoantigens (**Table 2**).

#### 2.2.1 CD19

Human CD19 antigen belongs to type-I transmembrane glycoprotein of the IgG immunoglobulin superfamily. In normal tissues, CD19 is specifically expressed throughout the development of B-cell lineage except for hematopoietic stem cells and terminal plasma cells, whereas it is absent on other hematopoietic lineages. In B-cell malignancies, its expression is widely distributed in relapsed/refractory B-cell acute lymphoblastic leukemia (R/R B-ALL) and relapsed/refractory B-cell non-Hodgkin lymphoma (R/R B-NHL) (45). Despite low expression of CD19 on MM cells, CD19 is expressed on the minor multiple myeloma stem cell (MMSC) subset that has been reported (46). MMSC is capable of self-renewal and drug-resistance. Thus, CD19 might be a potential target for MM. One clinical trial (NCT02135406) indicated that autologous stem cell transplantation (ASCT) followed by CD19-targeted CAR-T therapy (CTL019) infusion was safe and available in RRMM, leading to a longer PFS compared to patients with ASCT alone (47, 48).

#### 2.2.2 SLAMF7

SLAMF7 belongs to the signaling lymphocyte activation molecule family (SLAMF). SLAMF7 is firstly documented in natural killer cells (49). It is also expressed on T cells, B cells, monocytes, macrophages, and dendritic cells. Over 95% of normal or malignant plasma cells of MM expressed SLAMF7 (50). Since SLAMF7 is also expressed in normal plasma cells, specific attacks on this target inevitably cause normal cell death. Thereby, SLAMF7 is an alternative but suboptimal choice for CAR-T cell therapy.

The function of SLAMF7 is poorly understood, but previous evidence indicates its similar role as growth factor contributing to myeloma cell proliferation (51, 52). It has been reported that SLAMF7-CAR T cells attack myeloma and confer selective fratricide of SLAMF7-positive normal lymphocytes (53). A conceivable side effect is the depletion of SLAMF7+ lymphocytes, including a substantial proportion of T cells, B cells, and NK cells. It would be reasonable to engineer SLAMF7-CAR T cells with a safety switch to terminate fratricide of normal lymphocytes. Inducible caspase 9 or herpes simplex virus thymidine kinase might be preferable choices for safety switch (54, 55).

Several anti-SLAMF7 CAR constricts are evaluated in clinical trials, mostly as monotherapy (NCT03710421, NCT04142619, NCT04541368, NCT03958656, NCT04499339), or as dual CARs targeting both BCMA and SLAMF7 (NCT04795882, NCT04156269).

#### 2.2.3 GPRC5D

The G protein-coupled receptor, class C group 5 member D (GPRC5D), is expressed on 98% of the CD138+ cells by quantitative immunofluorescence (56). Also, this surface receptor is primarily expressed on hair follicles, but also in multiple myeloma cells. Therefore, GPRC5D-targeted CAR-T was constructed by Smith et al., which displayed potent anti-MM effects on MM cell lines and xenografted models (56). Anti-GPRC5D was deemed safe and effective as no alopecia or any skin-related disorders were detected in a preclinical study (57). A series of GPRC5D-CAR T trials are ongoing, such as NCT05219721, NCT04555551, NCT05016778. MCARH109, as the first-in-class GPRC5D-targeted CAR T-cell therapy for MM, has a manageable safety profile and high rates of clinical response (ORR: 83%). More importantly, all 6 patients who relapsed after BCMA-targeted CAR-T responded to MCARH109.

#### 2.2.4 CD138

As a major extracellular matrix (ECM) receptor, CD138 (syndecan-1) plays an important role in cell-cell and cell-matrix adhesion, and cell proliferation (58, 59). CD138 is widely expressed on normal and malignant plasma cells (60), but also expressed on the surface of mature epithelial cells that might cause skin toxicity. A prior study found that a high concentration of CD138 might be poor prognostic factor for MM (61). A CD138-directed CAR-T (CART-138) has been built incorporating with a 4-1BB domain (62). Relevant CD138-targeted CAR trials include single-target (NCT01886976, NCT03672318, NCT03196414, NCT03778346) and multi-target CAR-T products (NCT03271632). Based on current data (NCT01886976), the ORR achieved 80% and no toxicity has been reported, manifesting a good efficacy and tolerability. However, CD138 shedding and skin toxicity are major barriers for wide application of CD138-targeted CAR-T.

#### 2.2.5 CD38

CD38, a transmembrane glycoprotein, is known to mediate cell adhesion, signal transduction, and Ca^2+^ regulation (63). CD38 is highly expressed on the surface of MM cells, though its expression in normal hematopoietic cells also have been detected, such as T cells, precursors of B cells, NK cells, and myeloid precursors (63). Some monoclonal antibodies against CD38 have been approved by FDA to treat multiple myeloma, such as Daratumumab. The success of mAb targeting CD38 in the treatment of MM has encouraged the development of CD38-targeted CAR T cells. Light-chain exchange technology brings potential to avoid accident damage to CD38^+^ normal cells (64). A clinical trial (NCT0346491) investigated CD38-targeted CAR-T as a monotherapy for RRMM. In addition, dual CAR products are also tested in clinical trials, combing CD38 and BCMA (NCT03767751), CD38 and CD19 (NCT03125577).

#### 2.2.6 CD70

Aberrant expression of CD70 has been found in hematological malignancies and solid tumors (65). Because of its limited expression on normal cells, CD70 holds great promises for monoclonal antibody-based therapy. A preclinical study supported that CD70-targeted CAR T-cell therapy was safe and effective (66). Further, related publications manifested that CD70 targeting CAR-T cells caused robust anti-tumor activity in both human cancer cells and animal models (67, 68). It is worth noting that a clinical trial (NCT04662294) on CD70 is recruiting RRMM patients, although no data has been reported yet. Importantly, an obvious advantage is a low risk of fratricidal killing caused by CD70 antibody, mainly because of the transient expression of CD70 on immune cells (8).

#### 2.2.7 NKG2DL

NKG2D, a cell surface receptor binding to several ligands, is predominantly expressed on immune cytotoxic cells, such as NK cell and CD8^+^ cytotoxic T cells. NKG2D ligands, such as MIC-A, MIC-B, and UL-16, are upregulated in many solid tumors or hematologic malignancies but absent on healthy tissue. NKG2D binds to corresponding ligands to prompt the secretion of proinflammatory cytokines and the activation of cytotoxic cells, leading to immune elimination of MM cells (69). Due to the presence of a natural costimulatory domain, DAP10, there is no need to add this specific domain to NKG2D CARs. But a potential challenge is the poor persistency of T cells. To resolve this problem, patients should be treated with high doses or multiple infusions without compromising the toxicity (70). Satisfactorily, higher doses have the same safety profile with low doses, with no reports of CRS or NTX so far. We have identified one NKG2D CAR study (NCT03018405) in MM with an enrollment of 12 patients, but efficacy profile has not been published.

#### 2.2.8 Kappa light chain

Although cell surface immunoglobulins are not expressed on all plasma cells, it is recognized that MM stem cells express surface immunoglobulins (71). Thereby, kappa light chain might be an ideal target for MM (71). Several monoclonal antibodies targeting kappa light chain have been developed and tested in clinical trials, such as MDX-1097 (72). But CAR-targeting kapa light chain is still a less explored field. In one trial conducted by Ramos et al., 4 of 7 RRMM patients responded to kappa-targeted CAR-T cell therapy, keeping disease stable for 2-17 months. In a phase-I trial of *κ*-CAR-T cells (NCT00881920), 16 patients with non-Hodgkin lymphoma/chronic lymphocytic leukemia or MM were enrolled. Notably, 4 of 7 patients with relapsed or refractory MM kept disease stable for 2-17 months (71).

## 3 Mechanisms of disease resistance/relapse after MM CAR-T

**Figure 3:**
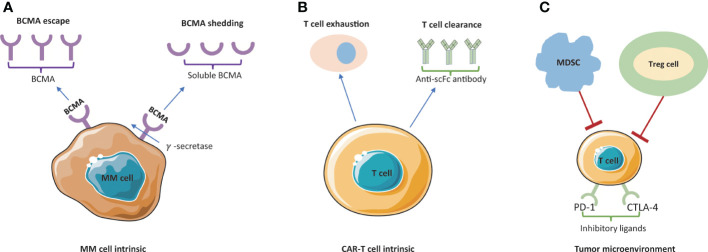
Mechanism of resistance/relapse to BCMA-targeted CAR-T cell therapy. **(A)** BCMA escape and BCMA shedding are blocking the antigen recognition by CAR-T cells. Membrane BCMA can be cleaved by γ–secretase and released to the plasma as soluble BCMA (sBCMA). **(B)** Poor persistence of CAR-T cells is mainly caused by T cell exhaustion and T cell clearance. **(C)** The tumor immunosuppressive microenvironment is mainly led by inhibitory ligands (PD-1 and CTLA-4) and suppressive immune cells (MDSC and Treg cells). MM, multiple myeloma; MDCS, myeloid-derived suppressor cells; Treg cell, T regulatory cells.

Despite the impressive ORR, over 50% of patients after BCMA-directed CAR-T would relapse or progress within 1-year (73). Another study showed a consistent preliminary trend that most MM patients who achieved MRD-negative to bb2121 have progressed in follow-up period (74). Thus, though CAR-T cells have the robust cytoreductive capacity to treat multiple myeloma, they cannot produce lasting immune surveillance. Currently, exact mechanism of disease resistance/relapse after MM CAR-T remains elusive, but there are several deductive mechanisms stated as following: 1) T cell-dependent resistance; 2) antigen-driven resistance (antigen escape, antigen shedding); 3) TME-related resistance. Some mechanisms are presented in **Figure 3**.

### 3.1 Poor persistence of CAR T cells

One study suggested that CAR-T cells were detectable up to 3 months after CAR-T injection and were gradually eliminated (73). At 12 months after infusion, only approximately 20% of patients had detectable engineered T cells (73). A lot of efforts have been made to figure out potential mechanisms leading to short persistence of CAR-T cells (73).

#### 3.1.1 T cell clearance

CAR-T cells are immunogenicity, thereby they might be eliminated by adaptive immune response over time. Single-chain fragment variable (ScFv) is the most common antigen-binding counterpart in CAR-T constructs. Most of ScFvs in BCMA-directed CAR-T are derived from non-human species (73), which induce immunogenicity and thereby potentially limit the T-cell persistence. In legend-2 study (75), anti-ScFv antibodies were detected in 7 of 17 MM patients after receiving bi-epitope BCMA-targeting CAR-T (LCAR-B38M), and 6 of them had decreased CAR-T cells and experienced tumor recurrence. More specifically, camelid-derived ScFvs were used to assemble LCAR-B38M, specifically targeting two different epitopes of BCMA on MM cell surface. There are agreements that non-human ScFv can induce immunological reaction to produce anti-CAR antibodies, which eventually lead to T-cell clearance and constitute a higher risk of relapse after CAR-T. This observation also highlights the importance of manufacturing humanized ScFV.

#### 3.1.2 Lack of memory characteristics

The differentiation stage of CAR-T cells affects their proliferation and survival, strongly correlating with their anti-tumor activity (76–78). The immunophenotype of T-cell used to manufacture CAR-T is considerably pivotal for T-cell persistence. Each subset of T cells possessed heterogeneity of proliferation and longevity (79). For example, naïve T-cells, stem memory T-cells, and central memory T-cells present the best proliferation capacity and delayed exhaustion or senescence (80). The enrichment of CD27^+^/CD45RO^-^/CD8^+^ T cells with memory-like features is correlated with long-term remission (81, 82). Also, a high percentage of cytotoxic CD8^+^ T cells with a naïve or stem memory characteristic are found to persist much longer and expand better *in vivo*, achieving superior outcomes after BCMA-targeted CAR-T treatment (23).

This view keeps in line with a previous finding that longer persistence of CAR-T cells *in vivo* expansion has been associated with better clinical remission and survival for recipient patients (83–86). One study also indicated that persistent CAR-T cells detected in peripheral blood tend to generate superior clinical response even among patients with high-grade diseases (87). Therefore, naïve cells and memory cells are important for CAR-T cell manufacture, mainly because they display sustained proliferation and longer persistence *in vivo*.

#### 3.1.3 Impaired T cell fitness

The quality of T cells also profoundly affects their life span *in vivo*. Notably, malignancy itself and chemotherapy-related myelosuppression could hamper T-cell fitness (88). When patients receive many lines of myeloma treatments, the composition of T cells would change over time. Furthermore, patients who underwent more lines of chemotherapies tended to have less early memory T cells *in vivo* (89).

#### 3.1.4 T cell exhaustion

T-cell exhaustion is another potential culprit, mainly because of constitutive antigen-independent tonic signaling by CAR-T. A variety of factors are able to induce tonic signaling to form activating clusters, leading to off-target activation and T-cell exhaustion (90, 91). Optimizing the CAR to limit antigen-independent tonic signaling and increase antigen-dependent recognition could be beneficial for T-cell persistence. In an anti-GPRC5D model of CAR-T, an IgG4/IgG2-derived spacer with modifications has been raised by Smith and colleagues, which might delay T-cell exhaustion (57).

### 3.2 Antigen escape and shedding

Antigen escape and shedding are the most common causes of the failure of CAR-T cell strategy. First, downregulation of tumor antigen reduces the CAR-T cell targeting ability, weakening the tumor-killing effects. Second, increased antigen shedding into a soluble form could negatively affect the efficacy of CAR-T therapy.

BCMA represents an important target. Theoretically, nearly all MM patients express BCMA irrespective of newly diagnosed or relapsed (38). It remains controversial about whether BCMA expression level is associated with the response rate to BCMA-directed CAR-T cells. However, loss of BCMA expression was suspected in post-treatment residual MM cells. Based on existing findings, there is a transient phenomenon that BCMA disappeared after initial response and subsequently remerged over MM progression (92). Though MM relapse is mainly caused by BCMA-positive clones, cases of recurrence led by BCMA-negative target cells have been noticed (22, 93). For example, a recent study pointed out that BCMA-negative was suspected in 3 of 71 patients at disease progression (94).

BCMA shedding from plasma cells is mediated by *γ*-secretase, producing the soluble-BCMA (sBCMA) that serves as a circulatory biomarker. Previous literatures have demonstrated that sBCMA is associated with the tumor burden and the prognosis (41, 95). High levels of soluble-BCMA might competitively bind to ScFv and consequently interfere the precise recognition of MM cells by CAR-T cells (96). Inhibitors of *γ*-secretase avoid BCMA shedding from MM cells and reduce the interference of soluble BCMA. Intriguingly, based on preclinical data, soluble BCMA does not affect the function of novel BCMA-CAR T *in vitro* and *in vivo* (37). Up to date, there is no clear clinical evidence that the level of sBCMA could negatively affect the efficacy of BCMA-targeted CAR-T therapy.

Likewise, high levels of soluble SLAMF7 are associated with a worse response to elotuzumab, along with a shorter survival (97). In addition, soluble CD38 could reduce the anti-MM response of daratumumab (98). However, as a seven-transmembrane protein, the likelihood of GPRC5D shedding into serum is low (57, 99). It is interesting to find that GPRC5D expression is independent from BCMA, therefore it might be an alternative target for relapsed MM patients after BCMA-directed therapy due to BCMA loss or shedding (57).

### 3.3 TME suppression

The tumor microenvironment (TME) plays a critical role in drug-resistance mechanism. CAR T cells need to overcome inhibitory signals and immunosuppressive cells existing in the TME. Immunosuppressive cells consist of T regulatory cells (Treg), B regulatory cells, myeloid-derived suppressor cells (MDSC), and plasmacytoid dendritic cells. These subsets may negatively affect the function of CAR-T cells (100–102). Besides, inaccessibility of MM cells by CAR-T cells forms another barrier. It is true MM cells generally reside in bone marrow microenvironment involving various cell types and extracellular matrix (ECM), which make CAR-T cells difficult to access MM (103). A recent study (104) about B-cell lymphoma reported a similar observation that many CD19-targted CAR-T cells did not successfully reach their target destination. Although mature CAR-T tracing method are still unmet needs, it is widely accepted that MM exploits immunosuppressive TME to block the efficacy of CAR-T cells and consequently lead to high risks of recurrence. PD1-PDL1 axis is another major cause of CAR T-cell dysfunction (105, 106). PD-1 expressed on activated T cells, is capable of binding with PD-L1 expressed by MM cells, eventually leading to exhausted state of T cells (107).

## 4 Strategies to improve the efficacy

**Figure 4:**
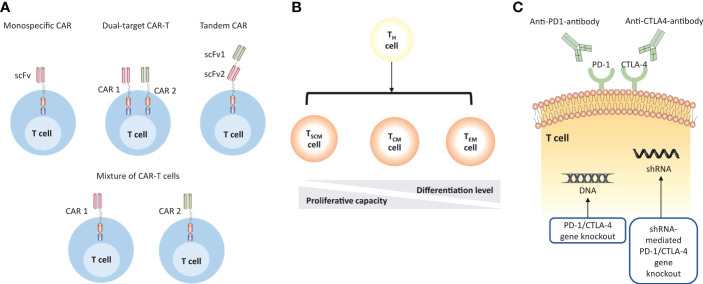
Strategies to improve the efficacy of MM CAR-T cell therapy. **(A)** CAR-T cell products are designed to target multiple TAAs to overcome antigen escape. The monospecific CAR has a single scFv; the dual-target CAR construct includes two separate monospecific CARs on the surface of T cells; the tandem CAR has two antigen-binding domains that are linked tandemly on one CAR protein; the mixture of CAR-T cells describes the simultaneous transduction of different types of CAR-T cells *in vivo*. **(B)** The persistence of CAR-T cells can be enhanced by using less-differentiated T cell subsets. **(C)** CAR-T cells can be engineered to overcome the immunosuppressive microenvironment by using immune checkpoint inhibitors or direct gene knockout.

For RRMM patients, poor persistence of T cells, antigen escape, and TME suppression restrict the durability of immune response and consequently limit the efficacy of CAR-T therapy in clinical settings. However, recently initiated studies have incorporated innovations to address above barriers (**Figure 4**, **Table 3**).

**Table 3:** Mechanisms of resistance to MM CAR-T and strategies to overcome the resistance.

	Resistance Mechanism		Strategies	Clinical Trial
**T-cell intrinsic**	Poor persistence	T cell clearance due to Immunogenicity	Manufacturing humanized ScFv with decreased immunogenicity	NCT03602612
		Lack of memory characteristics	Memory T cell-enriched product • Culture with PI3K inhibitors • Transduction with stem-cell memory T cell • CAR constructs with specific CD4:CD8 ratio	NCT03274219 NCT03288493 NCT03338972
		Impaired T cell fitness	• Allogeneic CAR-T cells • Receiving treatment at earlier MM stage	NCT04093596 NCT04196491
		T cell exhaustion	Limit antigen-independent tonic signaling and increase antigen-dependent recognition	NA
**MM intrinsic**	Antigen Escape	BCMA escape BCMA shedding	• Dual-/Multi-target design • Increased BCMA expression with gamma-secretase inhibitors	NCT04935580 NCT03502577
**TME**	Inhibitory signals and immunosuppressive cells	PD1-PDL1-mediated T cell dysregulation Immunosuppressive cells: Treg, MDSC	• Combined with immune checkpoint inhibitors • Combined with immunomodulatory drugs	NA

MM, multiple myeloma; TME, tumor microenvironment; Treg, T regulatory cells; MDSC, myeloid-derived suppressor cells; NA, not available.

### 4.1 Enhancing CAR-T cell persistence

Optimizing CAR-T design is a potential strategy to enhance CAR-T cell persistence. The utility of fully human recognition domains, rather than those derived from mouse antibodies, is an attempt to reduce immunogenicity which usually leads to clearance of CAR-T cells by patients’ immune system (22, 44, 75, 108, 109). Importantly, this strategy not only improves CAR-T cell persistence, but also simultaneously reduces cytokine storm. Besides, several studies demonstrated that the transmembrane region and co-stimulatory domain confer different properties of CAR-T cells that may influence efficacy and toxicity as well (110–125).

Another promising approach is to use less-differentiated T cells subsets that have a good proliferative capacity, such as naïve T cells, stem cell memory T cells (TSCM), central memory T cells (TCM). According to preclinical studies, CAR-T cells with memory phenotype presented superior engraftment, proliferation, and longevity compared to general CAR-T components (126, 127). Further, those who are treated with a defined ratio (1:1) of CD4^+^/CD8^+^ CAR-T cells, were monitored with more potent T cell expansion and fewer toxicities *in vivo* (128, 129).

In addition, lymphodepleting regimen may enhance the expansion of adoptively transferred T cells leading to superior persistence (130). First, lymphocytes depletion therapy before CAR-T could greatly reduce the risk of anti-CAR immune response. Second, a lymphodepleting environment is suitable for CAR-T cell expansion and persistence (80). It is known that IL-7 could assist CD8^+^ cytotoxic T-cell to preserve a stem memory phenotype *in vivo* (131), which is critical for T-cell expansion. All these data support the conclusion that more intense lymphodepletion may induce better CAR-T persistence and expansion.

### 4.2 Countering antigen escape

Increasing the density of BCMA expression is a critical area to counter antigen escape. The use of *γ*-secretase inhibitor (GSI) is able to increase BCMA expression on MM cells and reduce sBCMA levels by inhibiting the cleavage of surface BCMA (132). Preclinical models (133) have demonstrated that the presence of GSI could lead to a threefold to fivefold increase of BCMA expression level in MM cell lines. Particularly, when the density of BCMA is relatively low on the target cells, the administration of GSI may enhance the capacity of identifying MM cells. Great advancements in the efficacy of BCMA-targeted CAR T cells in combination with GSI have been observed in mouse models (133). Currently, several GSIs are being tested in clinical trials, even including patients with solid tumors (134). Future studies might discover other approaches to upregulate BCMA expression.

To address BCMA-negative clones, targeting two or more distinct antigens is underway. Due to the heterogeneity nature, targeting only one antigen at a time may not produce a long-lasting immunosurveillance in a large number of MM patients (135–137). More specifically, single target CAR-T only displays one single-chain variable fragment (ScFv) for antigen recognition, whereas dual-target CAR-T simultaneously contains co-stimulatory domain or tandem CAR molecules to overcome antigen escape and guarantee better identification. There are several strategies to achieve dual-target CAR-T products: 1) sequentially infusion of two CAR-T cells that respectively target different MM-associated antigen; 2) the same T cell displays two different CAR products; 3) One tandem CAR construct containing two antigen recognition moieties incorporated with one activation region (138). Available dual CAR products involved a combination of BCMA and CD19 (NCT04236011, NCT04162353), BCMA and SLAMF7 (NCT04662099, NCT04156269), BCMA and CD38 (NCT03767751). More details could be seen in **Table 4**.

**Table 4:** Dual-target or multi-target strategy tested in early clinical trials.

Antigen	Identifier	Status	Enrollment	Population
BCMA × CD19	NCT04935580	recruiting	20	NDMM, HRMM
BCMA × CD19	NCT04714827	recruiting	24	RRMM
BCMA × CD19	NCT04236011	recruiting	15	RRMM
BCMA × CD38	NCT03767751	recruiting	80	RRMM
BCMA × SLAMF7	NCT04156269	unknown	12	RRMM
BCMA × CD38 × CD138 × CD56	NCT03271632	recruiting	20	RRMM
BCMA × CD19 × CD38 × NYESO-1	NCT03638206	recruiting	73	RRMM

RRMM, relapsed or refractory multiple myeloma; HRMM, high risk multiple myeloma; NDMM, newly diagnosed multiple myeloma.

### 4.3 Overcoming immunosuppression in the TME

CAR-T cells should preliminarily overcome direct T cell inhibitory signals presented in the TME. PD1-PDL1 is the best characterized pathway. Inhibition of the PD-1 signals could produce dramatic clinical benefits in a variety types of tumors (139). Recent studies have demonstrated that coadministration of immune checkpoint inhibitors (ICI) with CAR-T therapy brought increased efficacy in preclinical models (105). In addition to ICI, knockout of the PD-1 coding gene could be engineered by gene silencing techniques, such as short hairpin RNAs (140) and CRISPR-Cas9 (141). Also, armoured CAR T cells secreting cytokines or chemokines are able to alter the inflammatory microenvironment and support the functionality of CAR T cells (142). Further, the metabolic competition between tumor and immune cells in the TME may restrict nutrient availability and cause microenvironment acidosis, which could trigger T cell inhibitory pathways or otherwise hinder immune cell function (143). Intriguingly, the expression of the antioxidant enzyme catalase in CAR-T cells may overcome granulocyte-mediated oxidative stress *in vitro* (144). Modifying T cell metabolism is a promising area to boost efficacy, but further validation is needed in clinical application.

## 5 Strategies to reduce the toxicity

Overall, treatment-related toxicity of MM CAR-T therapy involves two major categories: 1) general toxicity caused by T cell activation and following systemic cytokine storm; 2) specific toxicity caused by the interaction between CARs and TAAs expressed on non-tumor cells, which is also termed as ‘on-target, off-tumor’ toxicity.

### 5.1 Systemic cytokine storm

The rapid immune activation responsible for the success of CAR-T strategy also stimulates treatment-related toxicity. The clinical complications caused by different CAR-T in MM are similar to those led by CD19-targeted CAR-T in ALL and DLBCL (84, 145, 146), including cytokine release syndrome (CRS) and neurotoxicity (NTX), and hematologic cytopenia, which might limit the wide application of CAR-T cell therapy in MM.

The most frequent toxicity is cytokine release syndrome (CRS), a constellation of symptoms involving fever, myalgia, hypoxia and hypertension, resulting from increased inflammatory cytokines like IL-6. IL-6 receptor antagonism *via* Tocilizumab and short-course steroids could be used for CRS management (147). Besides, CAR-T cell-associated HLH/MAS is a more severe systemic hyperinflammatory syndrome. CAR-T cell-induced HLH/MAS may be resistant to IL-6 receptor inhibitors, of which condition chemotherapy would be required (145).

Neurotoxicity (NTX), is the second major adverse effect, mainly because of the disruption of the blood-brain barrier and increased cerebrospinal fluid cytokine levels (148). NTX frequently occurs with or following CRS, presenting encephalopathy, delirium, aphasia, seizures, and life-threatening cerebral oedema (149). The consensus grading scheme proposed by ASBMT was applied extensively (149). Notably, the grade 3-4 CRS and NTX could be effectively managed by tocilizumab and supportive care. Also, management of NTX comprises of corticosteroids and IL-6 pathway antagonisms (145). A special form of NTX is referred to immune effector cell-associated neurotoxicity syndrome (ICANS), as transient encephalopathy, which is attributed to off-target cytokine production, as well as immune response of central nervous system (CNS). A mounting evidence suggests that ICANS could be characterized by atypical features and prolonged timeframes (150). And its management coincides with CRS interventions, such as cytokine inhibitors and corticosteroids. However, current understanding of ICANS is still limited. The mechanisms for ICANS after BCMA-targeted therapy need further elucidation (151).

Hematologic cytopenia is commonly reported following BCMA CAR-T cell therapy, manifesting as leukopenia, lymphopenia, anemia, neutropenia, and thrombocytopenia, which could increase the risks of infection, bleeding, fever, and bruising (146, 152–154). After infusion, CAR-T cells not only activate tumor-specific T-cell, but also induce non-specific T or B clones that target hematopoietic stem cell (HSC), neutrophils, platelets, and erythroid cells (155). Besides, the release of cytokines could drive differentiation but arrest maturation of HSC (156). Therefore, the IL-6 blockade may control hematologic cytopenia as well. The management of cytopenia also includes transfusion of blood cells and growth factors of hematopoietic stem cell transplantation (HSCT) (157, 158).

**Figure 5:**
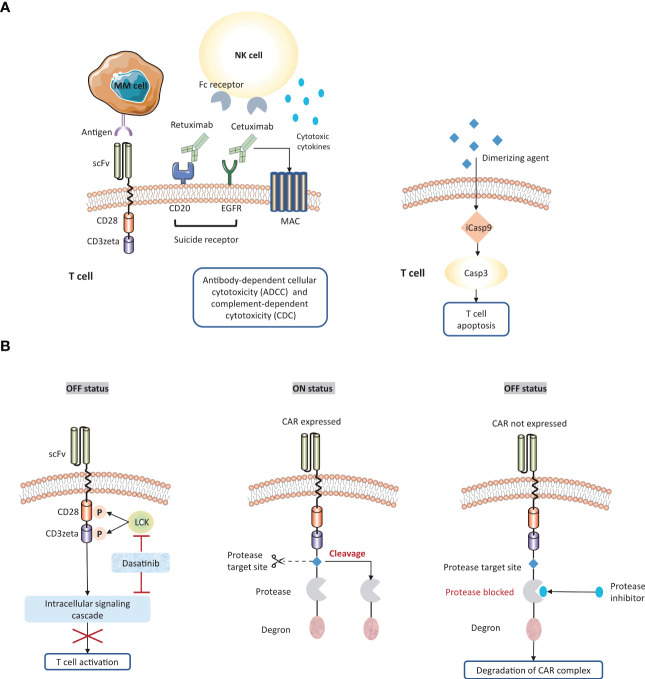
Strategies to overcome systemic cytokine toxicity. To counter systemic cytokine storm, several approaches are engineered to adjust CAR expression or activity. **(A)** Suicide gene system enables the elimination of CAR-T cells by following strategies: a) the activation of antibody-dependent cellular cytotoxicity (ADCC) or complement-dependent cytotoxicity (CDC); b) the induction of apoptosis pathway. **(B)** The ‘ON/OFF switches’ of CAR-T cells could be regulated by small molecular agents. scFv, single chain fragment variable; MAC, membrane attack complex; iCasp9, inducible caspase 9.

To counter systemic cytokine toxicity, CAR-T cells must reach a threshold level for activation but not exceed the level that would result in a series of cytokine secretion. Thus, therapeutic window for each CAR should be carefully considered. Researchers are currently engineering several innovations to control CAR expression or activity (**Figure 5**).

Firstly, 4-1BB co-stimulatory domain is associated with a much slower onset of T cell activation, increased T cell durability, and a lower risk of cytokine-related toxicity compared to CD28 domain. Therefore, inclusion of 4-1BB co-stimulatory domains might be less toxic in patients with heavy tumor burden. But CD28 is necessary to achieve the required threshold for T cell activation, especially for MM with a relatively low density of antigen or a low-affinity antigen-binding domain. Overall, the choice of co-stimulatory domain is critical to balance the efficacy and safety in CAR-T cell therapy.

Secondly, engineering ‘suicide genes’ into the CAR construct could induce apoptosis to eliminate CAR-T cells when treatment-related toxicity occurs. Co-expression of suicide receptors on MM CAR-T cells, such as CD20 and EGFR, could be attacked by rituximab (159–161) and cetuximab (162), respectively. Therefore, these FDA-approved antibodies provide a mean to deactivate CAR-T cells. Another strategy is to incorporate apoptosis-triggering fusion protein into CAR-T cells. iCasp9 is a well-characterized example, which can be triggered by dimerizing agents and subsequently drive rapid T cell depletion (163).

Thirdly, administration of small-molecular agents could control ‘on or off switch’ on CAR-T cells. Dasatinib, a tyrosine kinase inhibitor for CML and ALL. This agent enables the inhibition of LCK or intracellular signaling cascade, followed by destroying the downstream signal of activated CD3zeta. It has been demonstrated that dasatinib rapidly and reversibly hinder CAR-T cell activation, which provides a well-tolerated pharmacological toxicity switch without eradication of T cells (164). Alternatively, switch-off CARs (SMASh-CARs) provide another strategy to dynamically regulate T cell functionality *via* embedding a protease target, a protease, and a degron moiety (165). In the ‘OFF’ state, the degron moiety promotes the degradation of CAR-protease-degron complex. Protease inhibitors may function as the similar role to retain the degron structure. In the ‘On’ state, the protease target site is cleavage by protease leading to the removal of the degron from CAR protein, and consequently the CAR is expressed on the surface of T cells.

In addition, a more direct antagonism way is knockout of cytokine genes or expression of cytokine antagonists, both of which might provide opportunities to avert systemic toxicities. For example, the macrophage-activating and monocyte-activating cytokine GM-CSF can be antagonized by mutational inactivation and antibody lenzilumab, both of which can increase CAR T cell persistence while decreasing the risk of CRS.

### 5.2 On-target, off-tumor toxicity

Typically, CAR T cells are designed to target tumor-associated antigens (TAA). However, some TAAs are also expressed on the normal cells, leading to mistaken recognition and attack by CAR T cells. BCMA is a prominent TAA for CAR-T cell therapy in MM. However, the public transcriptomic datasets confirmed BCMA RNA expression in the caudate of normal human brains (166), indicating an on-target effect of anti-BCMA CAR-T therapy. Given the reports of phase-II cilta-cel study, 12 of 97 patients were reported with non-ICANS neurotoxicity. 5 of 97 (5.2%) patients suffered from a cluster of movement and neurocognitive symptoms (3 with ≥ Grade 3 parkinsonism) (167). Among them, one patient developed a progressive movement disorder with symptoms of parkinsonism around three months after BCMA-targeted CAR-T cell infusion. By analyzing this case, one study demonstrated that BCMA expression on neurons and astrocytes in the basal ganglia (166). Therefore, BCMA-targeted CAR-T cells may hold the potential to cross the blood-brain barrier and induce a progressive neurocognitive or movement disorder by targeting the basal ganglia. Close monitoring of neurotoxicity is necessary in patients with BCMA-targeted CAR-T cell therapies.

**Figure 6:**
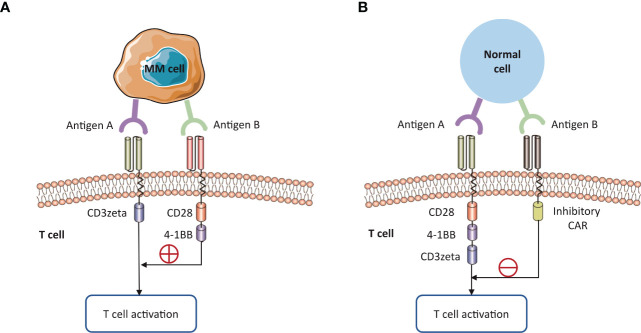
Strategies to overcome on-target, off-tumor toxicity. The expression of tumor-associated antigens on healthy tissues can lead to ‘on-target, off-tumor’ toxicity. **(A)** The specificity of CAR T cells is enhanced by targeting multiple TAAs. The activation domain and co-stimulatory domain should respectively bind to different antigens on MM cells for CAR T cell activation. **(B)** Alternative strategy is to use the inhibitory CAR against a specific non-tumor antigen, requiring the absence of this antigen on MM cells.

Engineering strategies aims to overcome on-target, off-tumor toxicity mediated by CAR-T cell therapy (**Figure 6**). The first strategy is to enhance the specificity of antigen recognition. Targeting multiple TAA is a promising approach. Specifically, CAR protein could be disassembled into two separate receptors, one with CD3zeta domain and another with a co-stimulatory domain. Both receptors need to recognize different TAAs for CAR T cells activation. Preclinical models have observed the promises in such a strategy (168–170). Alternatively, the inhibitory CAR (iCAR) contains a special inhibitory region that is generally derived from immune checkpoint proteins, such as PD-1 and CTLA-4. The inhibitory signal could recognize an antigen expressed on healthy tissues but absent on tumor cells (171). Moreover, engineering chimeric co-stimulatory receptor enables T cells to recognize antigens that are enriched on tumor cells. The second strategy is to utilize logic gating or conditional system to control CAR-T cell activation, such as the phospho-antigens that could be identified by T cell receptor. For example, HIF-1*α* degradation pathway is exploited to restrict CAR expression to CAR-T cells located in hypoxia TME, thereby avoiding adverse effects on healthy tissues which are normally non-hypoxic (172).

## 6 Innovations of MM CAR-T manufacture

Novel agents and CAR-T manufacture platforms are especially noteworthy. **Table 5** specifically focused on data of novel therapeutic agents for RRMM presented at major oncology meeting between 2020 and 2022, including Annual Society of Hematology (ASH) and American Society of Clinical Oncology (ASCO).

**Table 5:** Clinical trials of novel therapeutic agents for MM at recent oncology meetings, 2020-2022.

Product Name	Identifier	Target	Phase	Enrollment	Study Population	Country	Innovation	Clinical Update
P-BCMA-101	NCT03288493	BCMA	I-II	43	RRMM	United States	Using transposon-based system to enrich early memory T cells	ASH 2020 (173)
Orva-cel	NCT03430011 (EVOLVE)	BCMA	I-II	62	RRMM	United States	Fully human binder	ASCO 2020 (174)
JNJ-4528	NCT03548207 (CARTITUDE-1)	BCMA	I-II	17	RRMM	United States, Japan	A CAR-T therapy containing two BCMA-targeting single-domain antibodies	ASCO 2020 (175)
ALLO-715	NCT04093596 (UNIVERSAL)	BCMA	I	47	RRMM	United States	Allogeneic CAR-T product; Using TALEN technology to disrupt TCR constant gene	ASH 2021 (176)
CT053	NCT03975907 (LUMMICAR)	BCMA	I-II	14	RRMM	China	A fully human autologous CAR-T product	ASH 2021 (177)
ARI0002H	NCT04309981	BCMA	I-II	35	RRMM	Spanish	A lentiviral autologous second-generation CAR-T product	ASH 2021 (178)
PHE885	NCT04318327	BCMA	I	56	RRMM	United States	A novel CAR construct with an innovative T-charge manufacturing platform	ASH 2021 (179)
CT103A	ChiCTR1800018137	BCMA	I-II	71	RRMM	China	A fully human BCMA-specific CAR-T product	ASH 2021 (180)
bb2121	NCT03361748 (KarMMa)	BCMA	II	140	RRMM	Multicenter	Updated data of KarMMa trial	ASCO 2021 (181)
bb2121	NCT04196491 (KarMMa-4)	BCMA	I	13	NDMM	United States	Aiming at high-risk newly diagnosed MM patients	ASCO 2021 (24)
GC012F	NCT04236011	BCMA × CD19	I	28	RRMM	China	Rapid manufacture platform	ASCO 2022 (182)
CART-ddBCMA	NCT04155749	BCMA	I	25	RRMM	United States	An autologous CAR-T product that utilizes a novel, synthetic binding domain	ASCO 2022 (183)
OriCAR-017	NCT05016778	GPRC5D	I	11	RRMM	China	A novel CAR-T product with improvement in expansion and durability	ASCO 2022 (184)
Cilta-cel	NCT04133636 (CARTITUDE-2)	BCMA	II	19	RRMM	Multicenter	Update and supplement of CARTITUDE-1	ASCO 2022 (185)

RRMM, relapsed or refractory multiple myeloma; NDMM, newly diagnosed multiple myeloma; MM, multiple, myeloma; ASCO, American society of clinical oncology; ASH, American society of hematology.

### 6.1 Role of allogeneic CAR-T

Currently, all FDA-approved CAR-T constructs are manufactured within autologous T cells isolated from the patients’ blood. However, this individualized production process is somewhat costly and time-consuming, limiting the number of MM patients who can benefit from CAR-T therapy. First, the manufacture time of autological CAR-T cells is lengthy. Many patients with advanced stage of MM may be unable to benefit from this therapy (84, 186). Second, the production failure may be attributable to the insufficient T cells obtained from MM patients, as patients who previously received chemotherapy tend to undergo bone marrow suppression and lymphodepletion (88, 187). Third, the heterogeneity of apheresis CAR product is another underlying cause of preparation failure. There is a phenomenon that dysfunctional T subsets could result in inferior CAR-T products, consequently leading to poor efficacy and response rates (81, 188–191).

Allogeneic donor T cells provides an alternative to autological CAR-T cell therapy, which might potentially solve the manufacturing issues of inadequate T-cell number and suboptimal T-cell fitness for CAR-T production. CAR-T cells could be derived from HLA-matched allogeneic hemopoietic stem cell donors. Nevertheless, allogeneic CAR-T cell therapy has been associated with graft-versus-host (GVHD) and graft rejection. The engrafted allogeneic donor cells could launch an attack on recipient cells (192), whereas the host immune cells are able to eliminate allogeneic CAR-T cells. Recently, genetic modifications are explored to cope with T cell alloreactivity, such as TCR disruption and safety switch insertions (176). Genome-editing technologies include ZFN, TALEN, and CRISPR-Cas 9, all of which are used to generate universal CAR-T cells (193). Ongoing clinical trials provide novel armamentarium for MM immunotherapy.

At ASH 2021, updated results of an open-label, phase-I clinical study (UNIVERSAL, NCT04093596) were reported to validate the feasibility of allogeneic anti-BCMA ALLO-715 for RRMM (176). ALLO-715 is a genetically modified anti-BCMA CAR-T product which employed TALEN technology to disrupt the TCR constant gene and CD52 gene to prevent GVHD and allow the use of anti-CD52 based lymphodepletion (194). At the time of data cut-off, 47 patients were enrolled; 42 patients received ALLO-715 infusion. Efficacy outcomes presented 61.5% ORR among patients with high doses. Safety prolife showed CRS occurred in 52.4% and there was no grade 4-5 CRS. Overall, the UNIVERSAL trial demonstrates the proof for allogeneic CAR-T therapy for MM, which might bring meaningful efficacy and tolerable toxicity. But this trial continues to enroll more patients and follow-up data will be updated in the future (176).

### 6.2 Rapid CAR-T manufacture platform

In the process of commercial manufacture, patients need to wait for around 3-4 weeks until CAR-T infusion, in whom disease might progress while waiting for CAR production. The first-in-human dual BCMA and CD19 targeted CAR was manufactured by a novel platform (FAST CAR platform) that significantly reduced the production time to only 24-36 hours (195). Latest results of this trial (NCT04236011) showed a high response with 100% (DL-1: 1×10^5^/kg), 80% (DL-1: 2×10^5^/kg), and 93.8% (DL-1: 3×10^5^/kg) ORR, respectively. Also, 23 out of 28 patients (82.1%) suffered from grade 1-2 CRS and 2 patients (7.1%) with grade 3. The data presented promising efficacy and favorable safety of the BCMA-CD19 dual fast CAR-T for RRMM patients (182). This clinical trial is still ongoing and recruiting more patients.

At ASH 2021, a rapid manufacturing process that could both preserve the stemness of T cells to ensure longer durability and provide timely access for patients with aggressive disease, has been presented (28). Researchers developed a superior anti-BCMA CAR-T construct (PHE885) carrying a fully human anti-BCMA ScFv fused to 4-1BB/CD3_zeta_ signaling domains and an innovative T-Charge manufacturing platform, which enables rapid and reliable patient access. More specifically, this novel manufacturing platform allows PHE885 to preserve a higher percentage of naïve/T_SCM_ cells, leading to effectively engraft, expand, and reject tumors. Based on this principle, a phase-I trial (NCT04318327) has been initiated and early data of this study will be presented in the future.

### 6.3 Modified manufacturing process to harvest early memory T-cell

CAR-T cells start to disappear at first 3-6 months after infusion, subsequently leading to the loss of disease control. An innovation is to enrich early memory T cells by modification of manufacturing process. JCARH125 is a well elaborated example. Its production is optimized to harvest early memory T-cell and increase T-cell fitness. Relevant clinical trial (EVOLVE) data have been previously presented in ASH 2018. According to the latest reporting at ASCO 2020 (174), a total of 44 patients who received higher doses (300 × 10^6^, 450 × 10^6^, 600 × 10^6^) respectively achieved the ORR of 95%, 94%, and 71%. A promising finding is that functional CAR-T cells could be detected in 69% of cases at 6 months. P-BCMA-101 is an autologous BCMA-targeted CAR construct that consisted of a large number of stem cell memory cells. P-BCMA-101 was manufactured by a novel virus-free transposon “piggy-Bac” technology that preferentially transfect early memory T cells (196), thereby increasing efficacy while minimizing toxicity (173). A phase I-II study of P-BCMA-101 (NCT03288493) is being tested in RRMM patients and early data were reported in ASH 2018 (197). Current clinical data keep consistent with preclinical findings that the modifications of CAR production appear to have notably improved efficacy.

## 7 Conclusion

In this review, we summarized the current status and future innovations in CAR-T therapy for multiple myeloma. Clinical benefits of using CAR-T therapy to treat MM has been confirmed, but it does not lead to favorable durability and safety with current technologies. Numerous promising engineering approaches are underway to improve the efficacy and safety of CAR-T cell therapy, expanding this technology for a wider range of application and bring more benefits for MM patients.

## Author contributions

YQ and YJ designed the study and reviewed the manuscript. ZW and CC participated in study design and wrote the original draft of the manuscript. ZW and CC was mainly responsible for the design of tables and figures. LW contributed to the conception of the paper. All authors contributed to the article and approved the submitted version.

## References

[1] Kehrer M Koob S Strauss A Wirtz DC Schmolders J. [Multiple myeloma - current status in diagnostic testing and therapy]. Z fur Orthopadie und Unfallchirurgie (2017) 155(5):575–86. doi: 10.1055/s-0043-110224 28806822

[2] Michels TC Petersen KE. Multiple myeloma: Diagnosis and treatment. Am Family Physician (2017) 95(6):373–83. 28318212

[3] Padala SA Barsouk A Barsouk A Rawla P Vakiti A Kolhe R. Epidemiology, staging, and management of multiple myeloma. Med Sci (Basel Switzerland) (2021) 9(1):3. doi: 10.3390/medsci9010003 PMC7838784 33498356

[4] Kumar SK Rajkumar V Kyle RA van Duin M Sonneveld P Mateos MV. Multiple myeloma. Nat Rev Dis Primers (2017) 3:17046. doi: 10.1038/nrdp.2017.46 28726797

[5] Casey M Nakamura K. The cancer-immunity cycle in multiple myeloma. ImmunoTargets Ther (2021) 10:247–60. doi: 10.2147/itt.S305432 PMC8291851 34295843

[6] Kegyes D Constantinescu C Vrancken L Rasche L Gregoire C Tigu B. Patient selection for car T or bite therapy in multiple myeloma: Which treatment for each patient? J Hematol Oncol (2022) 15(1):78. doi: 10.1186/s13045-022-01296-2 35672793 PMC9171942

[7] Abramson HN. The multiple myeloma drug pipeline-2018: A review of small molecules and their therapeutic targets. Clin lymphoma Myeloma Leukemia (2018) 18(9):611–27. doi: 10.1016/j.clml.2018.06.015 30001985

[8] Wu C Zhang L Brockman QR Zhan F Chen L. Chimeric antigen receptor T cell therapies for multiple myeloma. J Hematol Oncol (2019) 12(1):120. doi: 10.1186/s13045-019-0823-5 31752943 PMC6873434

[9] Ye CJ Chen J Liu G Heng HH. Somatic genomic mosaicism in multiple myeloma. Front Genet (2020) 11:388. doi: 10.3389/fgene.2020.00388 32391059 PMC7189895

[10] Heng J Heng HH. Genome chaos: Creating new genomic information essential for cancer macroevolution. Semin Cancer Biol (2022) 81:160–75. doi: 10.1016/j.semcancer.2020.11.003 33189848

[11] Kumar SK Dimopoulos MA Kastritis E Terpos E Nahi H Goldschmidt H. Natural history of relapsed myeloma, refractory to immunomodulatory drugs and proteasome inhibitors: A multicenter imwg study. Leukemia (2017) 31(11):2443–8. doi: 10.1038/leu.2017.138 28620163

[12] Kochenderfer JN Somerville RPT Lu T Yang JC Sherry RM Feldman SA. Long-duration complete remissions of diffuse Large b cell lymphoma after anti-Cd19 chimeric antigen receptor T cell therapy. Mol Therapy: J Am Soc Gene Ther (2017) 25(10):2245–53. doi: 10.1016/j.ymthe.2017.07.004 PMC5628864 28803861

[13] Park JH Rivière I Gonen M Wang X Sénéchal B Curran KJ. Long-term follow-up of Cd19 car therapy in acute lymphoblastic leukemia. New Engl J Med (2018) 378(5):449–59. doi: 10.1056/NEJMoa1709919 PMC6637939 29385376

[14] Choi T Kang Y. Chimeric antigen receptor (Car) T-cell therapy for multiple myeloma. Pharmacol Ther (2022) 232:108007. doi: 10.1016/j.pharmthera.2021.108007 34582835 PMC8930424

[15] van der Stegen SJ Hamieh M Sadelain M. The pharmacology of second-generation chimeric antigen receptors. Nat Rev Drug Discovery (2015) 14(7):499–509. doi: 10.1038/nrd4597 26129802 PMC6410718

[16] Jensen MC Riddell SR. Designing chimeric antigen receptors to effectively and safely target tumors. Curr Opin Immunol (2015) 33:9–15. doi: 10.1016/j.coi.2015.01.002 25621840 PMC4397136

[17] Benmebarek MR Karches CH Cadilha BL Lesch S Endres S Kobold S. Killing mechanisms of chimeric antigen receptor (Car) T cells. Int J Mol Sci (2019) 20(6):1283. doi: 10.3390/ijms20061283 30875739 PMC6470706

[18] Sadelain M Rivière I Riddell S. Therapeutic T cell engineering. Nature (2017) 545(7655):423–31. doi: 10.1038/nature22395 PMC5632949 28541315

[19] Srivastava S Riddell SR. Engineering car-T cells: Design concepts. Trends Immunol (2015) 36(8):494–502. doi: 10.1016/j.it.2015.06.004 26169254 PMC4746114

[20] Lim WA June CH. The principles of engineering immune cells to treat cancer. Cell (2017) 168(4):724–40. doi: 10.1016/j.cell.2017.01.016 PMC5553442 28187291

[21] Brudno JN Kochenderfer JN. Recent advances in car T-cell toxicity: Mechanisms, manifestations and management. Blood Rev (2019) 34:45–55. doi: 10.1016/j.blre.2018.11.002 30528964 PMC6628697

[22] Brudno JN Maric I Hartman SD Rose JJ Wang M Lam N. T Cells genetically modified to express an anti-B-Cell maturation antigen chimeric antigen receptor cause remissions of poor-prognosis relapsed multiple myeloma. J Clin Oncology: Off J Am Soc Clin Oncol (2018) 36(22):2267–80. doi: 10.1200/jco.2018.77.8084 PMC6067798 29812997

[23] Cohen AD Garfall AL Stadtmauer EA Melenhorst JJ Lacey SF Lancaster E. B cell maturation antigen-specific car T cells are clinically active in multiple myeloma. J Clin Invest (2019) 129(6):2210–21. doi: 10.1172/jci126397 PMC6546468 30896447

[24] Usmani SZ Berdeja JG Truppel-Hartmann A Fei Y Wortman-Vayn H Shelat S. Karmma-4: Idecabtagene vicleucel (Ide-cel, Bb2121), a bcma-directed car T-cell therapy in high-risk newly diagnosed multiple myeloma. J Clin Oncol (2021) 39(15_suppl):TPS8053–TPS. doi: 10.1200/JCO.2021.39.15_suppl.TPS8053

[25] Kumar SK Baz RC Orlowski RZ Anderson LD Jr. Ma H Shrewsbury A. Results from lummicar-2: A phase 1b/2 study of fully human b-cell maturation antigen-specific car T cells (Ct053) in patients with relapsed and/or refractory multiple myeloma. Blood (2020) 136(Supplement 1):28–9. doi: 10.1182/blood-2020-139802

[26] Jie J Hao S Jiang S Li Z Yang M Zhang W. Phase 1 trial of the safety and efficacy of fully human anti-bcma car T cells in Relapsed/Refractory multiple myeloma. Blood (2019) 134(Supplement_1):4435. doi: 10.1182/blood-2019-126104

[27] An G Sui W Wang T Qu X Zhang X Yang J. An anti-bcma car T-cell therapy (C-Car088) shows promising safety and efficacy profile in relapsed or refractory multiple myeloma. Blood (2020) 136(Supplement 1):29–30. doi: 10.1182/blood-2020-138734

[28] Sperling AS Nikiforow S Nadeem O Mo CC Laubach JP Anderson KC. Phase I study of Phe885, a fully human bcma-directed car-T cell therapy for Relapsed/Refractory multiple myeloma manufactured in &Lt;2 days using the T-charge Tm platform. Blood (2021) 138(Supplement 1):3864. doi: 10.1182/blood-2021-146646

[29] Han L Gao Q Zhou K Zhou J Yin Q-S Fang B. The clinical study of anti-bcma car-T with single-domain antibody as antigen binding domain. J Clin Oncol (2021) 39(15_suppl):8025. doi: 10.1200/JCO.2021.39.15_suppl.8025

[30] Liu Y Chen Z Wei R Shi L He F Shi Z. Remission observed from a phase 1 clinical study of car-T therapy with safety switch targeting bcma for patients with Relapsed/Refractory multiple myeloma. J Clin Oncol (2018) 36(15_suppl):8020. doi: 10.1200/JCO.2018.36.15_suppl.8020

[31] Zhao WH Wang BY Chen LJ Fu WJ Xu J Liu J. Four-year follow-up of lcar-B38m in relapsed or refractory multiple myeloma: A phase 1, single-arm, open-label, multicenter study in China (Legend-2). J Hematol Oncol (2022) 15(1):86. doi: 10.1186/s13045-022-01301-8 35794616 PMC9261106

[32] Madduri D Berdeja JG Usmani SZ Jakubowiak A Agha M Cohen AD. Cartitude-1: Phase 1b/2 study of ciltacabtagene autoleucel, a b-cell maturation antigen-directed chimeric antigen receptor T cell therapy, in Relapsed/Refractory multiple myeloma. Blood (2020) 136(Supplement 1):22–5. doi: 10.1182/blood-2020-136307

[33] Hao S Jin J Jiang S Li Z Zhang W Yang M. Two-year follow-up of investigator-initiated phase 1 trials of the safety and efficacy of fully human anti-bcma car T cells (Ct053) in Relapsed/Refractory multiple myeloma. Blood (2020) 136(Supplement 1):27–8. doi: 10.1182/blood-2020-140156

[34] Novak AJ Darce JR Arendt BK Harder B Henderson K Kindsvogel W. Expression of bcma, taci, and baff-r in multiple myeloma: A mechanism for growth and survival. Blood (2004) 103(2):689–94. doi: 10.1182/blood-2003-06-2043 14512299

[35] Roex G Timmers M Wouters K Campillo-Davo D Flumens D Schroyens W. Safety and clinical efficacy of bcma car-T-Cell therapy in multiple myeloma. J Hematol Oncol (2020) 13(1):164. doi: 10.1186/s13045-020-01001-1 33272302 PMC7713173

[36] Bu DX Singh R Choi EE Ruella M Nunez-Cruz S Mansfield KG. Pre-clinical validation of b cell maturation antigen (Bcma) as a target for T cell immunotherapy of multiple myeloma. Oncotarget (2018) 9(40):25764–80. doi: 10.18632/oncotarget.25359 PMC5995247 29899820

[37] Carpenter RO Evbuomwan MO Pittaluga S Rose JJ Raffeld M Yang S. B-cell maturation antigen is a promising target for adoptive T-cell therapy of multiple myeloma. Clin Cancer Res: an Off J Am Assoc Cancer Res (2013) 19(8):2048–60. doi: 10.1158/1078-0432.Ccr-12-2422 PMC3630268 23344265

[38] Seckinger A Delgado JA Moser S Moreno L Neuber B Grab A. Target expression, generation, preclinical activity, and pharmacokinetics of the bcma-T cell bispecific antibody Em801 for multiple myeloma treatment. Cancer Cell (2017) 31(3):396–410. doi: 10.1016/j.ccell.2017.02.002 28262554

[39] Tai YT Acharya C An G Moschetta M Zhong MY Feng X. April And bcma promote human multiple myeloma growth and immunosuppression in the bone marrow microenvironment. Blood (2016) 127(25):3225–36. doi: 10.1182/blood-2016-01-691162 PMC4920023 27127303

[40] Sanchez E Gillespie A Tang G Ferros M Harutyunyan NM Vardanyan S. Soluble b-cell maturation antigen mediates tumor-induced immune deficiency in multiple myeloma. Clin Cancer Res: an Off J Am Assoc Cancer Res (2016) 22(13):3383–97. doi: 10.1158/1078-0432.Ccr-15-2224 26960399

[41] Sanchez E Li M Kitto A Li J Wang CS Kirk DT. Serum b-cell maturation antigen is elevated in multiple myeloma and correlates with disease status and survival. Br J Haematol (2012) 158(6):727–38. doi: 10.1111/j.1365-2141.2012.09241.x 22804669

[42] Quinn J Glassford J Percy L Munson P Marafioti T Rodriguez-Justo M. April Promotes cell-cycle progression in primary multiple myeloma cells: Influence of d-type cyclin group and translocation status. Blood (2011) 117(3):890–901. doi: 10.1182/blood-2010-01-264424 20709908

[43] Lee L Bounds D Paterson J Herledan G Sully K Seestaller-Wehr LM. Evaluation of b cell maturation antigen as a target for antibody drug conjugate mediated cytotoxicity in multiple myeloma. Br J Haematol (2016) 174(6):911–22. doi: 10.1111/bjh.14145 27313079

[44] Ali SA Shi V Maric I Wang M Stroncek DF Rose JJ. T Cells expressing an anti-B-Cell maturation antigen chimeric antigen receptor cause remissions of multiple myeloma. Blood (2016) 128(13):1688–700. doi: 10.1182/blood-2016-04-711903 PMC5043125 27412889

[45] Wang K Wei G Liu D. Cd19: A biomarker for b cell development, lymphoma diagnosis and therapy. Exp Hematol Oncol (2012) 1(1):36. doi: 10.1186/2162-3619-1-36 23210908 PMC3520838

[46] Johnsen HE Bøgsted M Schmitz A Bødker JS El-Galaly TC Johansen P. The myeloma stem cell concept, revisited: From phenomenology to operational terms. Haematologica (2016) 101(12):1451–9. doi: 10.3324/haematol.2015.138826 PMC5479618 27903712

[47] Garfall AL Stadtmauer EA Hwang WT Lacey SF Melenhorst JJ Krevvata M. Anti-Cd19 car T cells with high-dose melphalan and autologous stem cell transplantation for refractory multiple myeloma. JCI Insight (2018) 3(8):e120505. doi: 10.1172/jci.insight.120505 29669947 PMC5931130

[48] Garfall AL Maus MV Hwang WT Lacey SF Mahnke YD Melenhorst JJ. Chimeric antigen receptor T cells against Cd19 for multiple myeloma. New Engl J Med (2015) 373(11):1040–7. doi: 10.1056/NEJMoa1504542 PMC4646711 26352815

[49] Boles KS Mathew PA. Molecular cloning of Cs1, a novel human natural killer cell receptor belonging to the Cd2 subset of the immunoglobulin superfamily. Immunogenetics (2001) 52(3-4):302–7. doi: 10.1007/s002510000274 11220635

[50] Hsi ED Steinle R Balasa B Szmania S Draksharapu A Shum BP. Cs1, a potential new therapeutic antibody target for the treatment of multiple myeloma. Clin Cancer Res: an Off J Am Assoc Cancer Res (2008) 14(9):2775–84. doi: 10.1158/1078-0432.Ccr-07-4246 PMC4433038 18451245

[51] Tai YT Dillon M Song W Leiba M Li XF Burger P. Anti-Cs1 humanized monoclonal antibody Huluc63 inhibits myeloma cell adhesion and induces antibody-dependent cellular cytotoxicity in the bone marrow milieu. Blood (2008) 112(4):1329–37. doi: 10.1182/blood-2007-08-107292 PMC2515112 17906076

[52] Kikuchi J Hori M Iha H Toyama-Sorimachi N Hagiwara S Kuroda Y. Soluble Slamf7 promotes the growth of myeloma cells *Via* homophilic interaction with surface Slamf7. Leukemia (2020) 34(1):180–95. doi: 10.1038/s41375-019-0525-6 31358854

[53] Gogishvili T Danhof S Prommersberger S Rydzek J Schreder M Brede C. Slamf7-car T cells eliminate myeloma and confer selective fratricide of Slamf7(+) normal lymphocytes. Blood (2017) 130(26):2838–47. doi: 10.1182/blood-2017-04-778423 29089311

[54] Bonini C Ferrari G Verzeletti S Servida P Zappone E Ruggieri L. Hsv-tk gene transfer into donor lymphocytes for control of allogeneic graft-Versus-Leukemia. Sci (New York NY) (1997) 276(5319):1719–24. doi: 10.1126/science.276.5319.1719 9180086

[55] Hoyos V Savoldo B Quintarelli C Mahendravada A Zhang M Vera J. Engineering Cd19-specific T lymphocytes with interleukin-15 and a suicide gene to enhance their anti-Lymphoma/Leukemia effects and safety. Leukemia (2010) 24(6):1160–70. doi: 10.1038/leu.2010.75 PMC2888148 20428207

[56] Smith EL Harrington K Staehr M Masakayan R Jones J Long T. T Cell therapy targeting G protein-coupled receptor class c group 5 member d (Gprc5d), a novel target for the immunotherapy of multiple myeloma. Blood (2018) 132(Supplement 1):589. doi: 10.1182/blood-2018-99-110471

[57] Smith EL Harrington K Staehr M Masakayan R Jones J Long TJ. Gprc5d is a target for the immunotherapy of multiple myeloma with rationally designed car T cells. Sci Trans Med (2019) 11(485):eaau7746. doi: 10.1126/scitranslmed.aau7746 PMC7508042 30918115

[58] Sanderson RD Turnbull JE Gallagher JT Lander AD. Fine structure of heparan sulfate regulates syndecan-1 function and cell behavior. J Biol Chem (1994) 269(18):13100–6. doi: 10.1016/S0021-9258(17)36804-7 8175735

[59] Wijdenes J Vooijs WC Clément C Post J Morard F Vita N. A plasmocyte selective monoclonal antibody (B-B4) recognizes syndecan-1. Br J Haematol (1996) 94(2):318–23. doi: 10.1046/j.1365-2141.1996.d01-1811.x 8759892

[60] Kambham N Kong C Longacre TA Natkunam Y. Utility of syndecan-1 (Cd138) expression in the diagnosis of undifferentiated malignant neoplasms: A tissue microarray study of 1,754 cases. Appl Immunohistochem Mol Morphol: AIMM (2005) 13(4):304–10. doi: 10.1097/01.pai.0000159773.50905.7b 16280658

[61] O’Connell FP Pinkus JL Pinkus GS. Cd138 (Syndecan-1), a plasma cell marker immunohistochemical profile in hematopoietic and nonhematopoietic neoplasms. Am J Clin Pathol (2004) 121(2):254–63. doi: 10.1309/617d-wb5g-nfwx-hw4l 14983940

[62] Guo B Chen M Han Q Hui F Dai H Zhang W. Cd138-directed adoptive immunotherapy of chimeric antigen receptor (Car)-modified T cells for multiple myeloma. J Cell Immunother (2016) 2(1):28–35. doi: 10.1016/j.jocit.2014.11.001

[63] Fernàndez JE Deaglio S Donati D Beusan IS Corno F Aranega A. Analysis of the distribution of human Cd38 and of its ligand Cd31 in normal tissues. J Biol Regul Homeostatic Agents (1998) 12(3):81–91. 9795836

[64] Drent E Themeli M Poels R de Jong-Korlaar R Yuan H de Bruijn J. A rational strategy for reducing on-target off-tumor effects of Cd38-chimeric antigen receptors by affinity optimization. Mol Therapy: J Am Soc Gene Ther (2017) 25(8):1946–58. doi: 10.1016/j.ymthe.2017.04.024 PMC5542711 28506593

[65] McEarchern JA Smith LM McDonagh CF Klussman K Gordon KA Morris-Tilden CA. Preclinical characterization of sgn-70, a humanized antibody directed against Cd70. Clin Cancer Res: an Off J Am Assoc Cancer Res (2008) 14(23):7763–72. doi: 10.1158/1078-0432.Ccr-08-0493 19047103

[66] Wang QJ Yu Z Hanada KI Patel K Kleiner D Restifo NP. Preclinical evaluation of chimeric antigen receptors targeting Cd70-expressing cancers. Clin Cancer Res: an Off J Am Assoc Cancer Res (2017) 23(9):2267–76. doi: 10.1158/1078-0432.Ccr-16-1421 PMC5411349 27803044

[67] Ge H Mu L Jin L Yang C Chang YE Long Y. Tumor associated Cd70 expression is involved in promoting tumor migration and macrophage infiltration in gbm. Int J Cancer (2017) 141(7):1434–44. doi: 10.1002/ijc.30830 28612394

[68] Jin L Ge H Long Y Yang C Chang YE Mu L. Cd70, a novel target of car T-cell therapy for gliomas. Neuro-oncology (2018) 20(1):55–65. doi: 10.1093/neuonc/nox116 28651374 PMC5761579

[69] Wensveen FM Jelenčić V Polić B. Nkg2d: A master regulator of immune cell responsiveness. Front Immunol (2018) 9:441. doi: 10.3389/fimmu.2018.00441 29568297 PMC5852076

[70] Nikiforow S Werner L Murad J Jacobs M Johnston L Patches S. Safety data from a first-in-Human phase 1 trial of Nkg2d chimeric antigen receptor-T cells in Aml/Mds and multiple myeloma. Blood (2016) 128(22):4052–. doi: 10.1182/blood.V128.22.4052.4052

[71] Ramos CA Savoldo B Torrano V Ballard B Zhang H Dakhova O. Clinical responses with T lymphocytes targeting malignancy-associated K light chains. J Clin Invest (2016) 126(7):2588–96. doi: 10.1172/jci86000 PMC4922690 27270177

[72] Hutchinson AT Jones DR Raison RL. Preclinical and clinical development of an anti-kappa free light chain mab for multiple myeloma. Mol Immunol (2015) 67(2 Pt A):89–94. doi: 10.1016/j.molimm.2015.04.013 25964097

[73] Raje N Berdeja J Lin Y Siegel D Jagannath S Madduri D. Anti-bcma car T-cell therapy Bb2121 in relapsed or refractory multiple myeloma. New Engl J Med (2019) 380(18):1726–37. doi: 10.1056/NEJMoa1817226 PMC8202968 31042825

[74] Raje NS Berdeja JG Lin Y Munshi NC Siegel DSD Liedtke M. Bb2121 anti-bcma car T-cell therapy in patients with Relapsed/Refractory multiple myeloma: Updated results from a multicenter phase I study. J Clin Oncol (2018) 36(15_suppl):8007. doi: 10.1200/JCO.2018.36.15_suppl.8007

[75] Xu J Chen LJ Yang SS Sun Y Wu W Liu YF. Exploratory trial of a biepitopic car T-targeting b cell maturation antigen in Relapsed/Refractory multiple myeloma. Proc Natl Acad Sci United States America (2019) 116(19):9543–51. doi: 10.1073/pnas.1819745116 PMC6510991 30988175

[76] Gattinoni L Lugli E Ji Y Pos Z Paulos CM Quigley MF. A human memory T cell subset with stem cell-like properties. Nat Med (2011) 17(10):1290–7. doi: 10.1038/nm.2446 PMC3192229 21926977

[77] Hinrichs CS Borman ZA Cassard L Gattinoni L Spolski R Yu Z. Adoptively transferred effector cells derived from naive rather than central memory Cd8+ T cells mediate superior antitumor immunity. Proc Natl Acad Sci United States America (2009) 106(41):17469–74. doi: 10.1073/pnas.0907448106 PMC2762661 19805141

[78] Gattinoni L Klebanoff CA Palmer DC Wrzesinski C Kerstann K Yu Z. Acquisition of full effector function in vitro paradoxically impairs the in vivo antitumor efficacy of adoptively transferred Cd8+ T cells. J Clin Invest (2005) 115(6):1616–26. doi: 10.1172/jci24480 PMC1137001 15931392

[79] Gattinoni L Speiser DE Lichterfeld M Bonini C. T Memory stem cells in health and disease. Nat Med (2017) 23(1):18–27. doi: 10.1038/nm.4241 28060797 PMC6354775

[80] McLellan AD Ali Hosseini Rad SM. Chimeric antigen receptor T cell persistence and memory cell formation. Immunol Cell Biol (2019) 97(7):664–74. doi: 10.1111/imcb.12254 31009109

[81] Fraietta JA Lacey SF Orlando EJ Pruteanu-Malinici I Gohil M Lundh S. Determinants of response and resistance to Cd19 chimeric antigen receptor (Car) T cell therapy of chronic lymphocytic leukemia. Nat Med (2018) 24(5):563–71. doi: 10.1038/s41591-018-0010-1 PMC6117613 29713085

[82] Gattinoni L Klebanoff CA Restifo NP. Paths to stemness: Building the ultimate antitumour T cell. Nat Rev Cancer (2012) 12(10):671–84. doi: 10.1038/nrc3322 PMC6352980 22996603

[83] Hsieh EM Scherer LD Rouce RH. Replacing car-T cell resistance with persistence by changing a single residue. J Clin Invest (2020) 130(6):2806–8. doi: 10.1172/jci136872 PMC7260020 32364534

[84] Maude SL Laetsch TW Buechner J Rives S Boyer M Bittencourt H. Tisagenlecleucel in children and young adults with b-cell lymphoblastic leukemia. New Engl J Med (2018) 378(5):439–48. doi: 10.1056/NEJMoa1709866 PMC5996391 29385370

[85] Porter DL Hwang WT Frey NV Lacey SF Shaw PA Loren AW. Chimeric antigen receptor T cells persist and induce sustained remissions in relapsed refractory chronic lymphocytic leukemia. Sci Trans Med (2015) 7(303):303ra139. doi: 10.1126/scitranslmed.aac5415 PMC5909068 26333935

[86] Kershaw MH Westwood JA Parker LL Wang G Eshhar Z Mavroukakis SA. A phase I study on adoptive immunotherapy using gene-modified T cells for ovarian cancer. Clin Cancer Res: an Off J Am Assoc Cancer Res (2006) 12(20 Pt 1):6106–15. doi: 10.1158/1078-0432.Ccr-06-1183 PMC2154351 17062687

[87] Louis CU Savoldo B Dotti G Pule M Yvon E Myers GD. Antitumor activity and long-term fate of chimeric antigen receptor–positive T cells in patients with neuroblastoma. Blood (2011) 118(23):6050–6. doi: 10.1182/blood-2011-05-354449 PMC3234664 21984804

[88] Das RK Vernau L Grupp SA Barrett DM. Naïve T-cell deficits at diagnosis and after chemotherapy impair cell therapy potential in pediatric cancers. Cancer Discovery (2019) 9(4):492–9. doi: 10.1158/2159-8290.Cd-18-1314 PMC6676489 30630850

[89] Dancy E Garfall AL Cohen AD Fraietta JA Davis M Levine BL. Clinical predictors of T cell fitness for car T cell manufacturing and efficacy in multiple myeloma. Blood (2018) 132(Supplement 1):1886. doi: 10.1182/blood-2018-99-115319

[90] Ajina A Maher J. Strategies to address chimeric antigen receptor tonic signaling. Mol Cancer Ther (2018) 17(9):1795–815. doi: 10.1158/1535-7163.Mct-17-1097 PMC6130819 30181329

[91] Hudecek M Sommermeyer D Kosasih PL Silva-Benedict A Liu L Rader C. The nonsignaling extracellular spacer domain of chimeric antigen receptors is decisive for in vivo antitumor activity. Cancer Immunol Res (2015) 3(2):125–35. doi: 10.1158/2326-6066.Cir-14-0127 PMC4692801 25212991

[92] Susanibar Adaniya SP Cohen AD Garfall AL. Chimeric antigen receptor T cell immunotherapy for multiple myeloma: A review of current data and potential clinical applications. Am J Hematol (2019) 94(S1):S28–33. doi: 10.1002/ajh.25428 30730071

[93] Green DJ Pont M Sather BD Cowan AJ Turtle CJ Till BG. Fully human bcma targeted chimeric antigen receptor T cells administered in a defined composition demonstrate potency at low doses in advanced stage high risk multiple myeloma. Blood (2018) 132(Supplement 1):1011. doi: 10.1182/blood-2018-99-117729

[94] Munshi NC Anderson LD Jr. Shah N Madduri D Berdeja J Lonial S. Idecabtagene vicleucel in relapsed and refractory multiple myeloma. New Engl J Med (2021) 384(8):705–16. doi: 10.1056/NEJMoa2024850 33626253

[95] Ghermezi M Li M Vardanyan S Harutyunyan NM Gottlieb J Berenson A. Serum b-cell maturation antigen: A novel biomarker to predict outcomes for multiple myeloma patients. Haematologica (2017) 102(4):785–95. doi: 10.3324/haematol.2016.150896 PMC5395119 28034989

[96] Chen CI Bahlis N Gasparetto C Tuchman SA Lipe BC Baljevic M. Selinexor, pomalidomide, and dexamethasone (Spd) in patients with relapsed or refractory multiple myeloma. Blood (2019) 134(Supplement_1):141. doi: 10.1182/blood-2019-122907

[97] Ishibashi M Soeda S Sasaki M Handa H Imai Y Tanaka N. Clinical impact of serum soluble Slamf7 in multiple myeloma. Oncotarget (2018) 9(78):34784–93. doi: 10.18632/oncotarget.26196 PMC6205184 30410677

[98] Nooka AK Kaufman JL Hofmeister CC Joseph NS Heffner TL Gupta VA. Daratumumab in multiple myeloma. Cancer (2019) 125(14):2364–82. doi: 10.1002/cncr.32065 30951198

[99] Kodama T Kochi Y Nakai W Mizuno H Baba T Habu K. Anti-Gprc5d/Cd3 bispecific T-Cell-Redirecting antibody for the treatment of multiple myeloma. Mol Cancer Ther (2019) 18(9):1555–64. doi: 10.1158/1535-7163.Mct-18-1216 31270154

[100] Oliva S Troia R D’Agostino M Boccadoro M Gay F. Promises and pitfalls in the use of pd-1/Pd-L1 inhibitors in multiple myeloma. Front Immunol (2018) 9:2749. doi: 10.3389/fimmu.2018.02749 30538704 PMC6277686

[101] Bonello F D’Agostino M Moscvin M Cerrato C Boccadoro M Gay F. Cd38 as an immunotherapeutic target in multiple myeloma. Expert Opin Biol Ther (2018) 18(12):1209–21. doi: 10.1080/14712598.2018.1544240 30394809

[102] Chauhan D Singh AV Brahmandam M Carrasco R Bandi M Hideshima T. Functional interaction of plasmacytoid dendritic cells with multiple myeloma cells: A therapeutic target. Cancer Cell (2009) 16(4):309–23. doi: 10.1016/j.ccr.2009.08.019 PMC2762396 19800576

[103] Hideshima T Mitsiades C Tonon G Richardson PG Anderson KC. Understanding multiple myeloma pathogenesis in the bone marrow to identify new therapeutic targets. Nat Rev Cancer (2007) 7(8):585–98. doi: 10.1038/nrc2189 17646864

[104] Cazaux M Grandjean CL Lemaître F Garcia Z Beck RJ Milo I. Single-cell imaging of car T cell activity in vivo reveals extensive functional and anatomical heterogeneity. J Exp Med (2019) 216(5):1038–49. doi: 10.1084/jem.20182375 PMC6504219 30936262

[105] Yoon DH Osborn MJ Tolar J Kim CJ. Incorporation of immune checkpoint blockade into chimeric antigen receptor T cells (Car-ts): Combination or built-in car-T. Int J Mol Sci (2018) 19(2):340. doi: 10.3390/ijms19020340 29364163 PMC5855562

[106] Moon EK Wang LC Dolfi DV Wilson CB Ranganathan R Sun J. Multifactorial T-cell hypofunction that is reversible can limit the efficacy of chimeric antigen receptor-transduced human T cells in solid tumors. Clin Cancer Res: an Off J Am Assoc Cancer Res (2014) 20(16):4262–73. doi: 10.1158/1078-0432.Ccr-13-2627 PMC4134701 24919573

[107] Gay F D’Agostino M Giaccone L Genuardi M Festuccia M Boccadoro M. Immuno-oncologic approaches: Car-T cells and checkpoint inhibitors. Clin lymphoma Myeloma Leukemia (2017) 17(8):471–8. doi: 10.1016/j.clml.2017.06.014 28689001

[108] Lam N Trinklein ND Buelow B Patterson GH Ojha N Kochenderfer JN. Anti-bcma chimeric antigen receptors with fully human heavy-Chain-Only antigen recognition domains. Nat Commun (2020) 11(1):283. doi: 10.1038/s41467-019-14119-9 31941907 PMC6962219

[109] Smith EL Staehr M Masakayan R Tatake IJ Purdon TJ Wang X. Development and evaluation of an optimal human single-chain variable fragment-derived bcma-targeted car T cell vector. Mol Therapy: J Am Soc Gene Ther (2018) 26(6):1447–56. doi: 10.1016/j.ymthe.2018.03.016 PMC5986730 29678657

[110] Shimasaki N Jain A Campana D. Nk cells for cancer immunotherapy. Nat Rev Drug Discovery (2020) 19(3):200–18. doi: 10.1038/s41573-019-0052-1 31907401

[111] Liu E Marin D Banerjee P Macapinlac HA Thompson P Basar R. Use of car-transduced natural killer cells in Cd19-positive lymphoid tumors. New Engl J Med (2020) 382(6):545–53. doi: 10.1056/NEJMoa1910607 PMC7101242 32023374

[112] Depil S Duchateau P Grupp SA Mufti G Poirot L. ‘Off-the-Shelf’ allogeneic car T cells: Development and challenges. Nat Rev Drug Discovery (2020) 19(3):185–99. doi: 10.1038/s41573-019-0051-2 31900462

[113] Ying Z Huang XF Xiang X Liu Y Kang X Song Y. A safe and potent anti-Cd19 car T cell therapy. Nat Med (2019) 25(6):947–53. doi: 10.1038/s41591-019-0421-7 PMC7518381 31011207

[114] Weinkove R George P Dasyam N McLellan AD. Selecting costimulatory domains for chimeric antigen receptors: Functional and clinical considerations. Clin Trans Immunol (2019) 8(5):e1049. doi: 10.1002/cti2.1049 PMC6511336 31110702

[115] Rezvani K. Adoptive cell therapy using engineered natural killer cells. Bone Marrow Transplant (2019) 54(Suppl 2):785–8. doi: 10.1038/s41409-019-0601-6 PMC7594488 31431708

[116] Salter AI Ivey RG Kennedy JJ Voillet V Rajan A Alderman EJ. Phosphoproteomic analysis of chimeric antigen receptor signaling reveals kinetic and quantitative differences that affect cell function. Sci Signaling (2018) 11(544):eaat6753. doi: 10.1126/scisignal.aat6753 PMC6186424 30131370

[117] Brudno JN Kochenderfer JN. Chimeric antigen receptor T-cell therapies for lymphoma. Nat Rev Clin Oncol (2018) 15(1):31–46. doi: 10.1038/nrclinonc.2017.128 28857075 PMC12145160

[118] Alabanza L Pegues M Geldres C Shi V Wiltzius JJW Sievers SA. Function of novel anti-Cd19 chimeric antigen receptors with human variable regions is affected by hinge and transmembrane domains. Mol Therapy: J Am Soc Gene Ther (2017) 25(11):2452–65. doi: 10.1016/j.ymthe.2017.07.013 PMC5675490 28807568

[119] Sabatino M Hu J Sommariva M Gautam S Fellowes V Hocker JD. Generation of clinical-grade Cd19-specific car-modified Cd8+ memory stem cells for the treatment of human b-cell malignancies. Blood (2016) 128(4):519–28. doi: 10.1182/blood-2015-11-683847 PMC4965906 27226436

[120] Geldres C Savoldo B Dotti G. Chimeric antigen receptor-redirected T cells return to the bench. Semin Immunol (2016) 28(1):3–9. doi: 10.1016/j.smim.2015.12.001 26797495 PMC5045286

[121] Zhao Z Condomines M van der Stegen SJC Perna F Kloss CC Gunset G. Structural design of engineered costimulation determines tumor rejection kinetics and persistence of car T cells. Cancer Cell (2015) 28(4):415–28. doi: 10.1016/j.ccell.2015.09.004 PMC5003056 26461090

[122] Teh BW Harrison SJ Worth LJ Spelman T Thursky KA Slavin MA. Risks, severity and timing of infections in patients with multiple myeloma: A longitudinal cohort study in the era of immunomodulatory drug therapy. Br J Haematol (2015) 171(1):100–8. doi: 10.1111/bjh.13532 26105211

[123] Blimark C Holmberg E Mellqvist UH Landgren O Björkholm M Hultcrantz M. Multiple myeloma and infections: A population-based study on 9253 multiple myeloma patients. Haematologica (2015) 100(1):107–13. doi: 10.3324/haematol.2014.107714 PMC4281323 25344526

[124] Xu Y Zhang M Ramos CA Durett A Liu E Dakhova O. Closely related T-memory stem cells correlate with in vivo expansion of Car.Cd19-T cells and are preserved by il-7 and il-15. Blood (2014) 123(24):3750–9. doi: 10.1182/blood-2014-01-552174 PMC4055922 24782509

[125] Zhang X Sun S Hwang I Tough DF Sprent J. Potent and selective stimulation of memory-phenotype Cd8+ T cells in vivo by il-15. Immunity (1998) 8(5):591–9. doi: 10.1016/s1074-7613(00)80564-6 9620680

[126] Petersen CT Hassan M Morris AB Jeffery J Lee K Jagirdar N. Improving T-cell expansion and function for adoptive T-cell therapy using ex vivo treatment with Pi3kδ inhibitors and vip antagonists. Blood Adv (2018) 2(3):210–23. doi: 10.1182/bloodadvances.2017011254 PMC5812323 29386194

[127] Morgan MA Schambach A. Engineering car-T cells for improved function against solid tumors. Front Immunol (2018) 9:2493. doi: 10.3389/fimmu.2018.02493 30420856 PMC6217729

[128] Turtle CJ Hanafi LA Berger C Hudecek M Pender B Robinson E. Immunotherapy of non-hodgkin’s lymphoma with a defined ratio of Cd8+ and Cd4+ Cd19-specific chimeric antigen receptor-modified T cells. Sci Trans Med (2016) 8(355):355ra116. doi: 10.1126/scitranslmed.aaf8621 PMC5045301 27605551

[129] Turtle CJ Hanafi LA Berger C Gooley TA Cherian S Hudecek M. Cd19 car-T cells of defined Cd4+:Cd8+ composition in adult b cell all patients. J Clin Invest (2016) 126(6):2123–38. doi: 10.1172/jci85309 PMC4887159 27111235

[130] Susanibar Adaniya S Stadtmauer EA Cohen AD. Car T cell therapy for multiple myeloma: What have we learned? Leukemia (2022) 36(6):1481–4. doi: 10.1038/s41375-022-01539-8 35351984

[131] Cieri N Camisa B Cocchiarella F Forcato M Oliveira G Provasi E. Il-7 and il-15 instruct the generation of human memory stem T cells from naive precursors. Blood (2013) 121(4):573–84. doi: 10.1182/blood-2012-05-431718 23160470

[132] Laurent SA Hoffmann FS Kuhn PH Cheng Q Chu Y Schmidt-Supprian M. Γ-secretase directly sheds the survival receptor bcma from plasma cells. Nat Commun (2015) 6:7333. doi: 10.1038/ncomms8333 26065893 PMC4490565

[133] Pont MJ Hill T Cole GO Abbott JJ Kelliher J Salter AI. Γ-secretase inhibition increases efficacy of bcma-specific chimeric antigen receptor T cells in multiple myeloma. Blood (2019) 134(19):1585–97. doi: 10.1182/blood.2019000050 PMC6871311 31558469

[134] Ran Y Hossain F Pannuti A Lessard CB Ladd GZ Jung JI. Γ-secretase inhibitors in cancer clinical trials are pharmacologically and functionally distinct. EMBO Mol Med (2017) 9(7):950–66. doi: 10.15252/emmm.201607265 PMC5494507 28539479

[135] Furukawa Y Kikuchi J. Molecular basis of clonal evolution in multiple myeloma. Int J Hematol (2020) 111(4):496–511. doi: 10.1007/s12185-020-02829-6 32026210

[136] Kumar SK Rajkumar SV. The multiple myelomas - current concepts in cytogenetic classification and therapy. Nat Rev Clin Oncol (2018) 15(7):409–21. doi: 10.1038/s41571-018-0018-y 29686421

[137] Bolli N Avet-Loiseau H Wedge DC Van Loo P Alexandrov LB Martincorena I. Heterogeneity of genomic evolution and mutational profiles in multiple myeloma. Nat Commun (2014) 5:2997. doi: 10.1038/ncomms3997 24429703 PMC3905727

[138] Yu S Yi M Qin S Wu K. Next generation chimeric antigen receptor T cells: Safety strategies to overcome toxicity. Mol Cancer (2019) 18(1):125. doi: 10.1186/s12943-019-1057-4 31429760 PMC6701025

[139] Postow MA Callahan MK Wolchok JD. Immune checkpoint blockade in cancer therapy. J Clin Oncology: Off J Am Soc Clin Oncol (2015) 33(17):1974–82. doi: 10.1200/jco.2014.59.4358 PMC4980573 25605845

[140] Cherkassky L Morello A Villena-Vargas J Feng Y Dimitrov DS Jones DR. Human car T cells with cell-intrinsic pd-1 checkpoint blockade resist tumor-mediated inhibition. J Clin Invest (2016) 126(8):3130–44. doi: 10.1172/jci83092 PMC4966328 27454297

[141] Rupp LJ Schumann K Roybal KT Gate RE Ye CJ Lim WA. Crispr/Cas9-mediated pd-1 disruption enhances anti-tumor efficacy of human chimeric antigen receptor T cells. Sci Rep (2017) 7(1):737. doi: 10.1038/s41598-017-00462-8 28389661 PMC5428439

[142] Chmielewski M Hombach AA Abken H. Of cars and trucks: Chimeric antigen receptor (Car) T cells engineered with an inducible cytokine to modulate the tumor stroma. Immunol Rev (2014) 257(1):83–90. doi: 10.1111/imr.12125 24329791

[143] Xu X Gnanaprakasam JNR Sherman J Wang R. A metabolism toolbox for car T therapy. Front Oncol (2019) 9:322. doi: 10.3389/fonc.2019.00322 31114756 PMC6503740

[144] Ando T Mimura K Johansson CC Hanson MG Mougiakakos D Larsson C. Transduction with the antioxidant enzyme catalase protects human T cells against oxidative stress. J Immunol (Baltimore Md: 1950) (2008) 181(12):8382–90. doi: 10.4049/jimmunol.181.12.8382 19050255

[145] Neelapu SS Tummala S Kebriaei P Wierda W Gutierrez C Locke FL. Chimeric antigen receptor T-cell therapy - assessment and management of toxicities. Nat Rev Clin Oncol (2018) 15(1):47–62. doi: 10.1038/nrclinonc.2017.148 28925994 PMC6733403

[146] Brudno JN Kochenderfer JN. Toxicities of chimeric antigen receptor T cells: Recognition and management. Blood (2016) 127(26):3321–30. doi: 10.1182/blood-2016-04-703751 PMC4929924 27207799

[147] Grupp SA Kalos M Barrett D Aplenc R Porter DL Rheingold SR. Chimeric antigen receptor-modified T cells for acute lymphoid leukemia. New Engl J Med (2013) 368(16):1509–18. doi: 10.1056/NEJMoa1215134 PMC4058440 23527958

[148] Santomasso BD Park JH Salloum D Riviere I Flynn J Mead E. Clinical and biological correlates of neurotoxicity associated with car T-cell therapy in patients with b-cell acute lymphoblastic leukemia. Cancer Discovery (2018) 8(8):958–71. doi: 10.1158/2159-8290.Cd-17-1319 PMC6385599 29880584

[149] Lee DW Santomasso BD Locke FL Ghobadi A Turtle CJ Brudno JN. Astct consensus grading for cytokine release syndrome and neurologic toxicity associated with immune effector cells. Biol Blood Marrow Transpl: J Am Soc Blood Marrow Transplant (2019) 25(4):625–38. doi: 10.1016/j.bbmt.2018.12.758 PMC12180426 30592986

[150] Mohyuddin GR Banerjee R Alam Z Berger KE Chakraborty R. Rethinking mechanisms of neurotoxicity with bcma directed therapy. Crit Rev Oncol/Hematol (2021) 166:103453. doi: 10.1016/j.critrevonc.2021.103453 34461271

[151] Garcia Borrega J Gödel P Rüger MA Onur ÖA Shimabukuro-Vornhagen A Kochanek M. In the eye of the storm: Immune-mediated toxicities associated with car-T cell therapy. HemaSphere (2019) 3(2):e191. doi: 10.1097/hs9.0000000000000191 31723828 PMC6746039

[152] Strati P Varma A Adkins S Nastoupil LJ Westin J Hagemeister FB. Hematopoietic recovery and immune reconstitution after axicabtagene ciloleucel in patients with Large b-cell lymphoma. Haematologica (2021) 106(10):2667–72. doi: 10.3324/haematol.2020.254045 PMC8485681 32732355

[153] Nahas GR Komanduri KV Pereira D Goodman M Jimenez AM Beitinjaneh A. Incidence and risk factors associated with a syndrome of persistent cytopenias after car-T cell therapy (Pctt). Leuk lymphoma (2020) 61(4):940–3. doi: 10.1080/10428194.2019.1697814 31793821

[154] Fried S Avigdor A Bielorai B Meir A Besser MJ Schachter J. Early and late hematologic toxicity following Cd19 car-T cells. Bone Marrow Transplant (2019) 54(10):1643–50. doi: 10.1038/s41409-019-0487-3 30809033

[155] Hegde M Joseph SK Pashankar F DeRenzo C Sanber K Navai S. Tumor response and endogenous immune reactivity after administration of Her2 car T cells in a child with metastatic rhabdomyosarcoma. Nat Commun (2020) 11(1):3549. doi: 10.1038/s41467-020-17175-8 32669548 PMC7363864

[156] Juluri KR Wu QV Voutsinas J Hou J Hirayama AV Mullane E. Severe cytokine release syndrome is associated with hematologic toxicity following Cd19 car T-cell therapy. Blood Adv (2022) 6(7):2055–68. doi: 10.1182/bloodadvances.2020004142 PMC9006285 34666344

[157] Zhang X Zhu L Zhang H Chen S Xiao Y. Car-T cell therapy in hematological malignancies: Current opportunities and challenges. Front Immunol (2022) 13:927153. doi: 10.3389/fimmu.2022.927153 35757715 PMC9226391

[158] Huang R Wang X Zhang X. Unity brings strength: Combination of car-T cell therapy and hsct. Cancer Lett (2022) 549:215721. doi: 10.1016/j.canlet.2022.215721 35537573

[159] Philip B Kokalaki E Mekkaoui L Thomas S Straathof K Flutter B. A highly compact epitope-based Marker/Suicide gene for easier and safer T-cell therapy. Blood (2014) 124(8):1277–87. doi: 10.1182/blood-2014-01-545020 24970931

[160] Griffioen M van Egmond EH Kester MG Willemze R Falkenburg JH Heemskerk MH. Retroviral transfer of human Cd20 as a suicide gene for adoptive T-cell therapy. Haematologica (2009) 94(9):1316–20. doi: 10.3324/haematol.2008.001677 PMC2738728 19734426

[161] Serafini M Manganini M Borleri G Bonamino M Imberti L Biondi A. Characterization of Cd20-transduced T lymphocytes as an alternative suicide gene therapy approach for the treatment of graft-Versus-Host disease. Hum Gene Ther (2004) 15(1):63–76. doi: 10.1089/10430340460732463 14965378

[162] Wang X Chang WC Wong CW Colcher D Sherman M Ostberg JR. A transgene-encoded cell surface polypeptide for selection, in vivo tracking, and ablation of engineered cells. Blood (2011) 118(5):1255–63. doi: 10.1182/blood-2011-02-337360 PMC3152493 21653320

[163] Di Stasi A Tey SK Dotti G Fujita Y Kennedy-Nasser A Martinez C. Inducible apoptosis as a safety switch for adoptive cell therapy. New Engl J Med (2011) 365(18):1673–83. doi: 10.1056/NEJMoa1106152 PMC3236370 22047558

[164] Mestermann K Giavridis T Weber J Rydzek J Frenz S Nerreter T. The tyrosine kinase inhibitor dasatinib acts as a pharmacologic on/Off switch for car T cells. Sci Trans Med (2019) 11(499):eaau5907. doi: 10.1126/scitranslmed.aau5907 PMC7523030 31270272

[165] Juillerat A Tkach D Busser BW Temburni S Valton J Duclert A. Modulation of chimeric antigen receptor surface expression by a small molecule switch. BMC Biotechnol (2019) 19(1):44. doi: 10.1186/s12896-019-0537-3 31269942 PMC6610870

[166] Van Oekelen O Aleman A Upadhyaya B Schnakenberg S Madduri D Gavane S. Neurocognitive and hypokinetic movement disorder with features of parkinsonism after bcma-targeting car-T cell therapy. Nat Med (2021) 27(12):2099–103. doi: 10.1038/s41591-021-01564-7 PMC8678323 34893771

[167] Berdeja JG Madduri D Usmani SZ Jakubowiak A Agha M Cohen AD. Ciltacabtagene autoleucel, a b-cell maturation antigen-directed chimeric antigen receptor T-cell therapy in patients with relapsed or refractory multiple myeloma (Cartitude-1): A phase 1b/2 open-label study. Lancet (London England) (2021) 398(10297):314–24. doi: 10.1016/s0140-6736(21)00933-8 34175021

[168] Lanitis E Poussin M Klattenhoff AW Song D Sandaltzopoulos R June CH. Chimeric antigen receptor T cells with dissociated signaling domains exhibit focused antitumor activity with reduced potential for toxicity in vivo. Cancer Immunol Res (2013) 1(1):43–53. doi: 10.1158/2326-6066.Cir-13-0008 24409448 PMC3881605

[169] Kloss CC Condomines M Cartellieri M Bachmann M Sadelain M. Combinatorial antigen recognition with balanced signaling promotes selective tumor eradication by engineered T cells. Nat Biotechnol (2013) 31(1):71–5. doi: 10.1038/nbt.2459 PMC5505184 23242161

[170] Wilkie S van Schalkwyk MC Hobbs S Davies DM van der Stegen SJ Pereira AC. Dual targeting of Erbb2 and Muc1 in breast cancer using chimeric antigen receptors engineered to provide complementary signaling. J Clin Immunol (2012) 32(5):1059–70. doi: 10.1007/s10875-012-9689-9 22526592

[171] Fedorov VD Themeli M Sadelain M. Pd-1- and ctla-4-Based inhibitory chimeric antigen receptors (Icars) divert off-target immunotherapy responses. Sci Trans Med (2013) 5(215):215ra172. doi: 10.1126/scitranslmed.3006597 PMC4238416 24337479

[172] Juillerat A Marechal A Filhol JM Valogne Y Valton J Duclert A. An oxygen sensitive self-decision making engineered car T-cell. Sci Rep (2017) 7:39833. doi: 10.1038/srep39833 28106050 PMC5247770

[173] Costello CL Cohen AD Patel KK Ali SS Berdeja JG Shah N. Phase 1/2 study of the safety and response of p-Bcma-101 car-T cells in patients with Relapsed/Refractory (R/R) multiple myeloma (Mm) (Prime) with novel therapeutic strategies. Blood (2020) 136(Supplement 1):29–30. doi: 10.1182/blood-2020-142695

[174] Mailankody S Jakubowiak AJ Htut M Costa LJ Lee K Ganguly S. Orvacabtagene autoleucel (Orva-cel), a b-cell maturation antigen (Bcma)-directed car T cell therapy for patients (Pts) with Relapsed/Refractory multiple myeloma (Rrmm): Update of the phase 1/2 evolve study (Nct03430011). J Clin Oncol (2020) 38(15_suppl):8504. doi: 10.1200/JCO.2020.38.15_suppl.8504

[175] Berdeja JG Madduri D Usmani SZ Singh I Zudaire E Yeh T-M. Update of cartitude-1: A phase Ib/Ii study of jnj-4528, a b-cell maturation antigen (Bcma)-directed car-T-Cell therapy, in Relapsed/Refractory multiple myeloma. J Clin Oncol (2020) 38(15_suppl):8505. doi: 10.1200/JCO.2020.38.15_suppl.8505

[176] Mailankody S Liedtke M Sidana S Matous JV Chhabra S Oluwole OO. Universal updated phase 1 data validates the feasibility of allogeneic anti-bcma allo-715 therapy for Relapsed/Refractory multiple myeloma. Blood (2021) 138(Supplement 1):651. doi: 10.1182/blood-2021-145572

[177] Chen W Fu C Cai Z Li Z Wang H Yan L. Sustainable efficacy and safety results from lummicar study 1: A phase 1/2 study of fully human b-cell maturation antigen-specific car T cells (Ct053) in Chinese subjects with relapsed and/or refractory multiple myeloma. Blood (2021) 138(Supplement 1):2821. doi: 10.1182/blood-2021-150124

[178] Fernandez de Larrea C Gonzalez-Calle V Cabañas V Oliver-Caldes A Español-Rego M Rodriguez-Otero P. Results from a pilot study of Ari0002h, an academic bcma-directed car-T cell therapy with fractionated initial infusion and booster dose in patients with relapsed and/or refractory multiple myeloma. Blood (2021) 138(Supplement 1):2837. doi: 10.1182/blood-2021-147188

[179] Bu D Bennett P Barton N Bradshaw L Pinon-Ortiz M Li X. Identification and development of Phe885: A novel and highly potent fully human anti-bcma car-T manufactured with a novel T-charge Tm platform for the treatment of multiple myeloma. Blood (2021) 138(Supplement 1):2770. doi: 10.1182/blood-2021-148390

[180] Li C Wang D Song Y Li J Huang H Chen B. A phase 1/2 study of a novel fully human b-cell maturation antigen-specific car T cells (Ct103a) in patients with relapsed and/or refractory multiple myeloma. Blood (2021) 138(Supplement 1):547. doi: 10.1182/blood-2021-152576

[181] Larry D Anderson J Munshi NC Shah N Jagannath S Berdeja JG. Idecabtagene vicleucel (Ide-cel, Bb2121), a bcma-directed car T cell therapy, in relapsed and refractory multiple myeloma: Updated karmma results. J Clin Oncol (2021) 39(15_suppl):8016. doi: 10.1200/JCO.2021.39.15_suppl.8016

[182] Du J Jiang H Dong B Gao L Liu L Ge J. Updated results of a multicenter first-in-Human study of Bcma/Cd19 dual-targeting fast car-T Gc012f for patients with Relapsed/Refractory multiple myeloma (Rrmm). J Clin Oncol (2022) 40(16_suppl):8005. doi: 10.1200/JCO.2022.40.16_suppl.8005

[183] Frigault MJ Rosenblatt J Cook D Cho HN Depinho GD Logan E. Phase 1 study of cart-ddbcma in relapsed or refractory multiple myeloma. J Clin Oncol (2022) 40(16_suppl):8003. doi: 10.1200/JCO.2022.40.16_suppl.8003

[184] Huang H Hu Y Zhang M Ding X Tang Y He X. Phase I open-label single arm study of Gprc5d car T-cells (Oricar-017) in patients with Relapsed/Refractory multiple myeloma (Polaris). J Clin Oncol (2022) 40(16_suppl):8004. doi: 10.1200/JCO.2022.40.16_suppl.8004

[185] Donk NWCJVD Agha ME Cohen AD Cohen YC Anguille S Kerre T. Biological correlative analyses and updated clinical data of ciltacabtagene autoleucel (Cilta-cel), a bcma-directed car-T cell therapy, in patients with multiple myeloma (Mm) and early relapse after initial therapy: Cartitude-2, cohort b. J Clin Oncol (2022) 40(16_suppl):8029. doi: 10.1200/JCO.2022.40.16_suppl.8029

[186] Schuster SJ Svoboda J Chong EA Nasta SD Mato AR Anak Ö. Chimeric antigen receptor T cells in refractory b-cell lymphomas. New Engl J Med (2017) 377(26):2545–54. doi: 10.1056/NEJMoa1708566 PMC5788566 29226764

[187] Singh N Perazzelli J Grupp SA Barrett DM. Early memory phenotypes drive T cell proliferation in patients with pediatric malignancies. Sci Trans Med (2016) 8(320):320ra3. doi: 10.1126/scitranslmed.aad5222 26738796

[188] Elavia N Panch SR McManus A Bikkani T Szymanski J Highfill SL. Effects of starting cellular material composition on chimeric antigen receptor T-cell expansion and characteristics. Transfusion (2019) 59(5):1755–64. doi: 10.1111/trf.15287 30973976

[189] Philip M Fairchild L Sun L Horste EL Camara S Shakiba M. Chromatin states define tumour-specific T cell dysfunction and reprogramming. Nature (2017) 545(7655):452–6. doi: 10.1038/nature22367 PMC5693219 28514453

[190] Schietinger A Philip M Krisnawan VE Chiu EY Delrow JJ Basom RS. Tumor-specific T cell dysfunction is a dynamic antigen-driven differentiation program initiated early during tumorigenesis. Immunity (2016) 45(2):389–401. doi: 10.1016/j.immuni.2016.07.011 27521269 PMC5119632

[191] Tötterman TH Carlsson M Simonsson B Bengtsson M Nilsson K. T-Cell activation and subset patterns are altered in b-cll and correlate with the stage of the disease. Blood (1989) 74(2):786–92. doi: 10.1182/blood.V74.2.786.786 2787679

[192] Kebriaei P Singh H Huls MH Figliola MJ Bassett R Olivares S. Phase I trials using sleeping beauty to generate Cd19-specific car T cells. J Clin Invest (2016) 126(9):3363–76. doi: 10.1172/jci86721 PMC5004935 27482888

[193] Zhao J Lin Q Song Y Liu D. Universal cars, universal T cells, and universal car T cells. J Hematol Oncol (2018) 11(1):132. doi: 10.1186/s13045-018-0677-2 30482221 PMC6257951

[194] Mailankody S Matous JV Liedtke M Sidana S Malik S Nath R. Universal: An allogeneic first-in-Human study of the anti-bcma allo-715 and the anti-Cd52 allo-647 in Relapsed/Refractory multiple myeloma. Blood (2020) 136(Supplement 1):24–5. doi: 10.1182/blood-2020-140641

[195] Jiang H Dong B Gao L Liu L Ge J He A. Clinical results of a multicenter study of the first-in-Human dual bcma and Cd19 targeted novel platform fast car-T cell therapy for patients with Relapsed/Refractory multiple myeloma. Blood (2020) 136(Supplement 1):25–6. doi: 10.1182/blood-2020-138614

[196] Hermanson DL Barnett BE Rengarajan S Codde R Wang X Tan Y. A novel bcma-specific, centyrin-based car-T product for the treatment of multiple myeloma. Blood (2016) 128(22):2127–. doi: 10.1182/blood.V128.22.2127.2127

[197] Gregory T Cohen AD Costello CL Ali SA Berdeja JG Ostertag EM. Efficacy and safety of p-Bcma-101 car-T cells in patients with Relapsed/Refractory (R/R) multiple myeloma (Mm). Blood (2018) 132(Supplement 1):1012. doi: 10.1182/blood-2018-99-111419

